# Cellular prion protein mediates early apoptotic proteome alternation and phospho-modification in human neuroblastoma cells

**DOI:** 10.1038/cddis.2016.384

**Published:** 2017-01-19

**Authors:** Saima Zafar, Christina Behrens, Hassan Dihazi, Matthias Schmitz, Inga Zerr, Walter J Schulz-Schaeffer, Sanja Ramljak, Abdul R Asif

**Affiliations:** 1 Department of Neurology, Georg-August University, Goettingen 37075, Germany; 2 Department of Neuropathology, Georg-August University, Goettingen 37075, Germany; 3 Department of Nephrology and Rheumatology, Georg-August University, Goettingen 37075, Germany; 4 Sciema UG, An der Hayl 4, Mainz 55130, Germany; 5 Institute for Clinical Chemistry / UMG-Laboratories, University Medical Center Goettingen, Georg-August University, Goettingen, Germany

## Abstract

Anti-apoptotic properties of physiological and elevated levels of the cellular prion protein (PrP^c^) under stress conditions are well documented. Yet, detrimental effects of elevated PrP^c^ levels under stress conditions, such as exposure to staurosporine (STS) have also been described. In the present study, we focused on discerning early apoptotic STS-induced proteome and phospho-proteome changes in SH-SY5Y human neuroblastoma cells stably transfected either with an empty or *PRNP*-containing vector, expressing physiological or supraphysiological levels of PrP^c^, respectively. PrP^c^-overexpression *per se* appears to stress the cells under STS-free conditions as indicated by diminished cell viability of PrP^c^-overexpressing *versus* control cells. However, PrP^c^-overexpression becomes advantageous following exposure to STS. Thus, only a short exposure (2 h) to 1 *μ*M STS results in lower survival rates and significantly higher caspase-3 activity in control *versus* PrP^c^-overexpressing cells. Hence, by exposing both experimental groups to the same apoptotic conditions we were able to induce apoptosis in control, but not in PrP^c^-overexpressing cells (as assessed by caspase-3 activity), which allowed for filtering out proteins possibly contributing to protection against STS-induced apoptosis in PrP^c^-overexpressing cells. Among other proteins regulated by different PrP^c^ levels following exposure to STS, those involved in maintenance of cytoskeleton integrity caught our attention. In particular, the finding that elevated PrP^c^ levels significantly reduce profilin-1 (PFN-1) expression. PFN-1 is known to facilitate STS-induced apoptosis. Silencing of PFN-1 expression by siRNA significantly increased viability of PrP^c^-overexpressing *versus* control cells, under STS treatment. In addition, PrP^c^-overexpressing cells depleted of PFN-1 exhibited increased viability *versus* PrP^c^-overexpressing cells with preserved PFN-1 expression, both subjected to STS. Concomitant increase in caspase-3 activity was observed in control *versus* PrP^c^-overexpressing cells after treatment with siRNA- PFN-1 and STS. We suggest that reduction of PFN-1 expression by elevated levels of PrP^c^ may contribute to protective effects PrP^c^-overexpressing SH-SY5Y cells confer against STS-induced apoptosis.

Apoptosis is essential for maintenance of cellular homeostasis as a part of normal development of the nervous system.^1^ At the same time apoptosis is also a characteristic of many neurodegenerative disorders.^2^ Furthermore, reduced apoptotic cell death or its obstruction is one of the critical cellular changes during malignant transformation.^3^

Considering that cellular prion protein (PrP^c^) is necessary for propagation of prion diseases and that apoptosis has been described in the brains of patients affected by these diseases,^4^ a more complete understanding of PrP^c^ impact on apoptotic cell death is required. Moreover, PrP^c^ appears to be involved in the pathogenesis of Alzheimer disease^5^ and in promoting invasiveness of different cancer cell types,^6, 7^ both of which are accompanied by dysregulated apoptosis.^3, 8^

Although expression of PrP^c^ at physiological levels is known to exert protective, anti-apoptotic effects *in vitro* as well as *in vivo,*^9, 10, 11^ other evidence strongly suggest that PrP^c^ overexpression in different cell lines sensitises cells to apoptotic stimuli.^12, 13^ It appears that an augmented susceptibility to apoptotic stimuli, such as staurosporine (STS), is governed by a p53-dependent pathway. Moreover, *in vivo* findings demonstrated that PrP^c^ overexpression can induce spontaneous neurodegeneration,^14, 15^ and that local PrP^c^ overexpression in muscles leads to primary myopathy, most likely via a p53 pathway.^16^ Earlier, we reported disturbed cellular homeostasis following PrP^c^ overexpression in human neuroblastoma SH-SY5Y cells, but were unable to show that a sole overexpression of PrP^c^ can alter p53 levels.^17^

Yet, another study employing mouse neuroblastoma N2a cell line suggested that physiological levels of PrP^c^ have a decisive protective role against STS-mediated cell death.^18^

Keeping in mind that elevated PrP^c^ levels *per se* may provoke neurodegeneration,^14^ that neurodegenerative diseases, including prion diseases are characterized by neuronal apoptosis,^19, 20^ and that rise in PrP^c^ expression promotes invasiveness and survival of cancer cells,^6, 7^ the aforementioned conflicting findings on PrP^c^ expression levels and its associated pro- and/or anti-apoptotic properties should be further elucidated.

This study aimed at revealing largely unknown proteome and phospho-proteome changes of early apoptotic events following treatment of human neuroblastoma SH-SY5Y control cells, stably overexpressing an empty vector, with apoptotic agent STS *versus* SH-SY5Y cells stably overexpressing PrP^c^ exposed to the same apoptotic agent.

STS is a non-selective protein kinase inhibitor that has been extensively used as one of the most potent pro-apoptotic stimuli in a variety of cells.^21, 22, 23^ Although molecular mechanisms of STS-induced apoptosis are still not completely clear an involvement of caspase activation^24^ is certain.

By identifying early changes in protein expression patterns between physiological and PrP^c^ overexpressing levels, on ‘the edge of apoptosis' (already present in control, but not in PrP^c^-overexpressing cells, as assessed by caspase-3 activation) we aimed at filtering out proteins contributing to previously observed expression level-mediated pro- and/or anti-apoptotic PrP^c^ properties. Identification of these candidate proteins might improve our understanding of PrP^c^ function both in health and disease.

## Results

**Figure 1:**
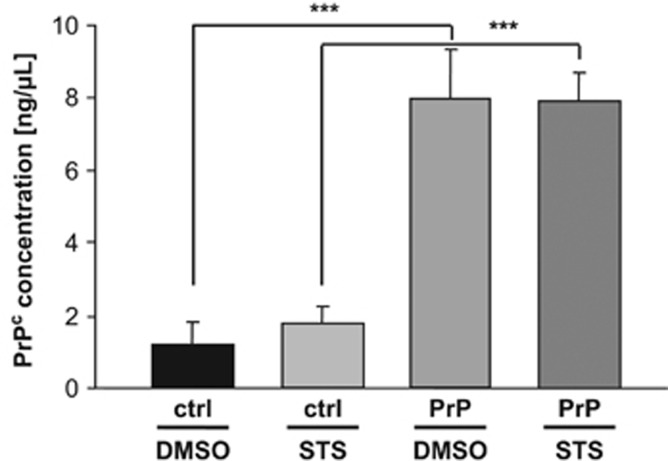
ELISA analysis of PrP^c^ levels in parental and SH-SY5Y cells stably transfected with pCIneo*PRNP* vector following DMSO/STS treatment. PrP^c^ levels were analyzed following treatment of parental (expressing endogenous PrP^c^; designated ctrl) and PrP^c^-overexpressing (designated PrP) SH-SY5Y cells with either DMSO or 1 *μ*M STS for 2 h. Note markedly, 7-and 5-fold, higher PrP^c^ expression in pCIneo*PRNP* transfected *versus* parental cells following DMSO and STS treatment, respectively. PrP^c^ concentration was measured in 50 *μ*g of proteins. Values represent the mean±S.D. of four independent experiments (two-sided unpaired Student's *t*-test ***P⩽0.001)

**Figure 2:**
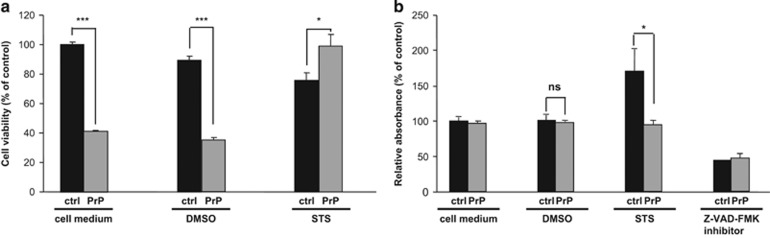
Cell viability (**a**) and caspase-3 activity (**b**) in empty vector transfected and PrP^c^-overexpressing SH-SY5Y cells subjected to apoptotic agent STS. SH-SY5Y cells expressing either endogenous PrP^c^ (designated ctrl/black bars) or overexpressing PrP^c^ (designated PrP/grey bars) were subjected to DMSO (vehicle control) or 1 *μ*M STS treatment for 2 h. As a negative control both, ctrl and PrP SH-SY5Y cells were incubated in the cell medium only, without addition of DMSO or STS. During performance of caspase assay a potent caspase-3 activity inhibitor (Z-VAD-FMK) was used as a positive control. Note, a significantly lower cell viability in PrP as compared with control cells under both, treatment-free (cell medium) and DMSO treatment conditions with no obvious changes in caspase-3 activity (ns). Conversely, after STS treatment a survival rate of PrP^c^-overexpressing cells was significantly higher as compared with cells expressing endogenous levels of PrP^c^. At the same time STS treatment resulted in caspase-3 activity in control, but not in PrP cells. Cell viability and caspase-3 activity were expressed as a percent of relative absorbance to untreated control cells expressing endogenous level of PrP^c^. Data are means±S.D. of duplicate measurements from four independent experiments. Level of significance was calculated using Student's *t*-test: *P⩽0.05; ***P⩽0.001

To identify early apoptotic changes following 2-h exposure to 1*μ*M STS a quantitative proteome and phospho-proteome analysis was performed using human neuroblastoma SH-SY5Y cells stably transfected either with *PRNP* or an empty vector, respectively. An introduction of pCIneo*PRNP* plasmid into SH-SY5Y cells treated with either DMSO or STS resulted in an average 5- (*P*=3.7 × 10^−5^) to 7-fold (*P*=6.5 × 10^−4^) higher expression of PrP^c^ in PrP^c^-overexpressing (designated PrP) *versus* control SH-SY5Y cells (designated ctrl), as quantified by ELISA measurements (Figure 1). Remarkably, PrP cells demonstrated diminished viability in MTS assay as compared with control cells, both under treatment-free conditions (*P*= 1 × 10^-5^) and following DMSO treatment (*P*= 6.5 × 10^−5^). After STS treatment an increased viability rates of PrP *versus* control cells were observed (*P*=0.019) (Figure 2a). Concomitantly, caspase-3 activity, a marker of programmed cell death, was significantly (*P*=0.017) increased in control as compared with PrP cells, both subjected to STS treatment (Figure 2b).

**Figure 3:**
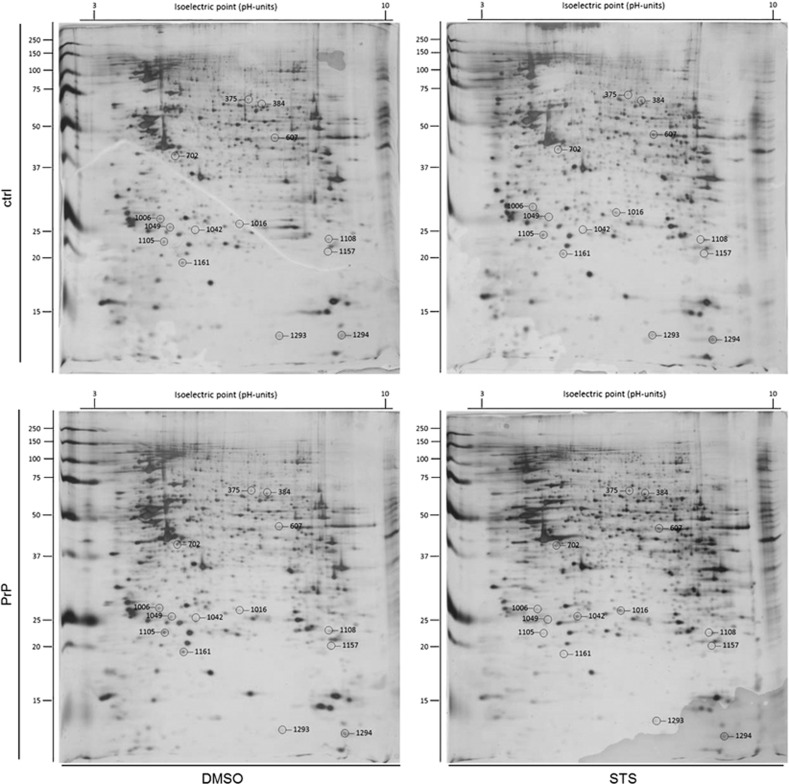
Silver-stained 2-DE gels of empty vector- and PrP^c^-overexpressing SH-SY5Y in the presence of DMSO/STS. Linear 17 cm IPG strips (pH 3-10) were used and loaded with 130 *μ*g of proteins. Labelling on the gel represents the location of 14 protein spots detected as differentially regulated between distinct transfection/treatment groups. Upper panels represent 2-DE gel pattern of SH-SY5Y cells stably transfected with an empty vector (designated ctrl), whereas lower panels represent the 2-DE pattern of SH-SY5Y cells stably overexpressing PrP^c^ (designated PrP). The panels on the left side depict 2-DE pattern following DMSO treatment, whereas panels on the right side depict 2-DE pattern following STS treatment. The protein identity of spots is listed in Table 1

**Figure 4:**
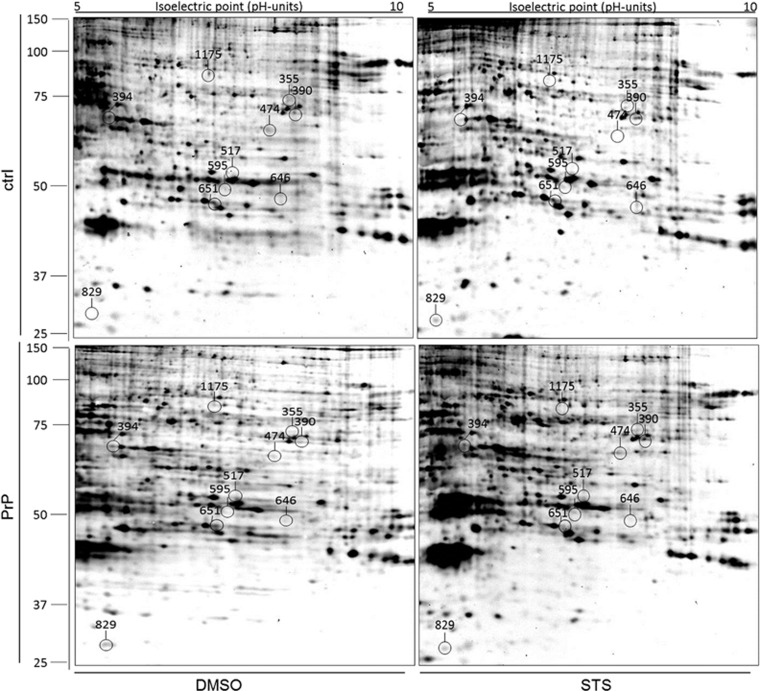
2-DE phospho-proteome of empty vector- and PrP^c^-overexpressing SH-SY5Y in the presence of DMSO/STS. Linear 17 cm IPG strips (pH 5-10) were used and loaded with 130* μ*g of proteins. Labelling on the gel represents the location of 11 protein spots detected as differentially regulated between distinct transfection/treatment groups (spot no. 394 remained unidentified). Upper panels show representative gels of control cells, expressing endogenous levels of PrP^c^, whereas lower panels show representative gels of PrP^c^-overexpressing SH-SY5Y cells. The panels on the left side: DMSO (vehicle control); the panels on the right side: STS treatment. The protein identity of spots is listed in Table 2

Densitometric analysis of silver-stained 2-DE gels revealed altogether 14 (Table 1), whereas phospho-protein staining revealed 10 (Table 2) differentially expressed proteins (Figures 3 and 4) across all compared experimental groups. To be able to differentiate between protein expression changes induced by either STS or the solvent (DMSO) six different experimental groups were compared (Tables 1 and 2).

The threshold for identification of up-/down-regulated proteins was set to 1.5- (proteome) and 1.4-fold (phospho-proteome) changes, respectively. Based on this criterion, we detected 11 up- and 10 down-regulated proteins (five distinct proteins were repeatedly found to be differentially regulated across different transfection/treatment groups) following silver staining of 2-DE gels throughout all experimental groups. Exemplarily, actin-interacting protein 1 was detected as differentially regulated in PrP^+STS^/ctrl^+STS^ and PrP^+STS^/ctrl^+DMSO^ group. Seven proteins exhibited a 2-fold or higher regulation after silver staining of 2-DE (Table 1). Two experimental groups: PrP^+DMSO^/ctrl^+STS^ and PrP^+STS^/ctrl^+DMSO^ did not demonstrate any differentially regulated proteins following silver staining.

**Figure 5:**
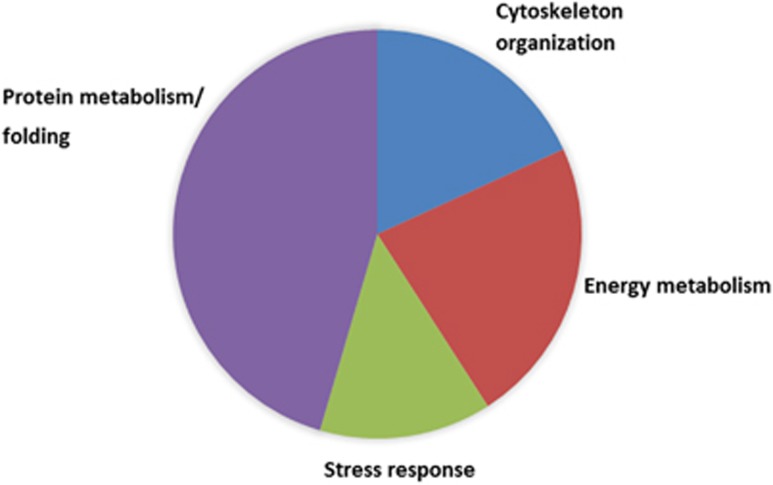
Distribution of all differentially regulated total and phospho-proteins in 2-DE in the form of functional categories

According to above mentioned criterion, we detected 4 up- and 7 down-regulated phosphorylated proteins throughout all experimental groups. Altogether, a majority of differentially expressed proteins (45.5%) appertains to a protein metabolism/folding group of proteins, 22.7% to energy metabolism, 18.2% of proteins belong to cytoskeleton group of proteins and finally 13.6% to stress response group (Figure 5).

**Figure 6:**
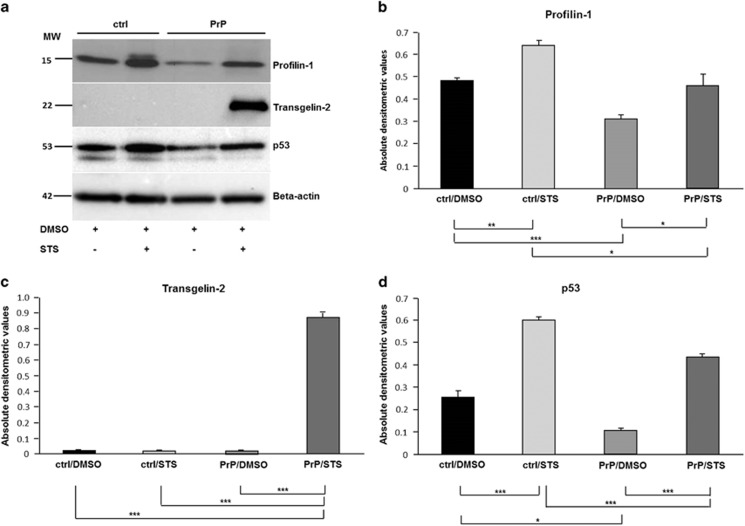
Profilin-1, transgelin-2 and p53 are differentially regulated between empty vector- and PrP^c^-overexpressing SH-SY5Y cells following DMSO/STS treatment. Western blot analysis shows a marked upregulation of profilin-1 in both empty vector (designated ctrl) and PrP^c^-overexpressing (designated PrP) SH-SY5Y cells subjected to STS as compared with DMSO treatment. An overexpression of PrP^c^ significantly decreases profilin-1 expression, regardless of DMSO/STS treatment. Transgelin-2 expression was detected only in cells overexpressing PrP^c^ subjected to STS treatment, whereas no signal was detected in PrP^c^-overexpressing cells subjected to DMSO. Likewise, no signal was obtained in control cells subjected either to DMSO or STS. p53 displays a similar expression pattern as profilin-1 with significantly higher expression in control as compared with PrP^c^-overexpressing cells under both experimental conditions. *β*-actin expression below is given as a control for an equal protein load. The displayed Western blots are representatives of three independent experiments (**a**). Expression level of each protein displayed by western blot was quantified by densitometric analysis and is shown as a diagram (**b**–**d**). Data were normalized against ß-actin and are given as a ratio of each protein/*β*-actin±S.D., *P⩽0.05; **P⩽0.01; ***P⩽0.001

Owing to the emerging role of PrP^c^ in maintenance of cytoskeleton organization,^25^ we decided to analyse expression level of two differentially regulated cytoskeletal proteins, PFN-1 and transgelin-2 using western blot analysis (Figure 6). Profilin-1 protein expression was 1.64-fold (*P*=0.042) lower in PrP^+STS^ *versus* ctrl^+STS^ group with a score of 627, whereas trangelin-2 expression was 3.65-fold (*P*=0.008) higher in PrP^+STS^ as compared with ctrl^+STS^ group with a score of 393 (Table 1). Densitometric analysis of western blot protein bands revealed ~1.5-fold lower expression of Pfn1 in PrP^+STS^ as compared with ctrl^+STS^ group (*P*=0.021). Nearly the same ratio of differential expression (1.5-fold) was retained between all the other groups compared, with the following levels of significance: ctrl^+DMSO^ *versus* ctrl^+STS^ (*P*=0.001); PrP^+DMSO^ *versus* PrP^+STS^ (*P*=0.046) and ctrl^+DMSO^ *versus* PrP^+DMSO^ (*P*=0.0002). A significant elevation of transgelin-1 expression is observed in PrP^+STS^ group as compared with all three other groups (*P*= 0.0003). Intriguingly, p53 expression pattern is analogues to that of Pfn1 (Figure 6a). Although not detected as regulated in densitometric analysis of 2-DE gels, we decided to check p53 expression levels between different experimental groups because of previously reported involvement of this protein in the exertion of PrP^c^ function when challenged with STS.^26^ Indeed, expression of p53 was 1.4-fold higher in ctrl^+STS^ *versus* PrP^+STS^ group (*P*=0.0003). Ctrl^+STS^ group showed 2.3-fold increase in p53 expression as compared with ctrl^+DMSO^ group (*P*= 0.0005) and 3.9-fold increase in PrP^+STS^ *versus* PrP^+DMSO^ group (*P*=7.2 × 10^−5^). Finally, a 2.3-fold decrease in p53 expression was observed in PrP^+DMSO^ as compared with ctrl^+DMSO^ group (*P*=0.010).

Owing to the fact that PFN-1 promotes STS-induced apoptosis,^27^ and elevated levels of PrP^c^ downregulate its expression in SH-SY5Y cells, we decided to examine the link between PrP^c^ and PFN-1 further.

**Figure 7:**
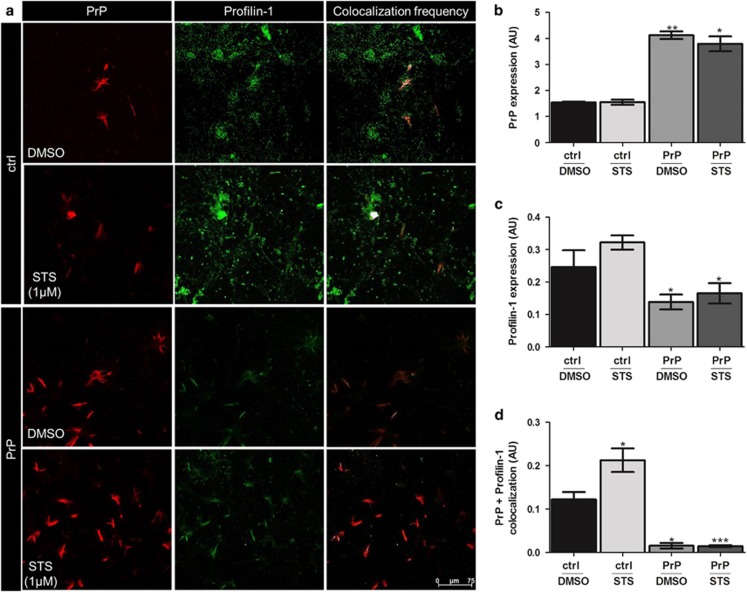
Co-localization of profilin-1 and PrP^c^ in SH-SY5Y cells following DMSO/STS treatment. Co-immunostaining of profilin-1 and PrP^c^ shows a marked altered localization of profilin-1 in both empty vector (designated ctrl) and PrP^c^-overexpressing (designated PrP) SH-SY5Y cells subjected to STS as compared with DMSO treatment (**a**). Quantification of images taken in different regions of SH-SY5Y cells fixed after STS treatment showed a significant re-distribution of profilin-1 in PrP-positive cells. Pearson's co-localization correlation coefficient rp (−1⩽ rp⩽1) and graph was generated by ImageJ (Bethesda, MD, USA) (WCIF plugin) software. Densitometric analysis was performed from four independent experiments and the level of significance was calculated using one-way ANOVA Friedman test: *P⩽0.05; **P⩽0.01; ***P⩽0.001 (**b**–**d**)

Immunofluorescence analysis additionally verified PrP^c^-mediated expressional regulation of PFN-1 as well as its altered co-localization pattern in ctrl^+STS^ *versus* PrP^+STS^ group (Figure 7).

**Figure 8:**
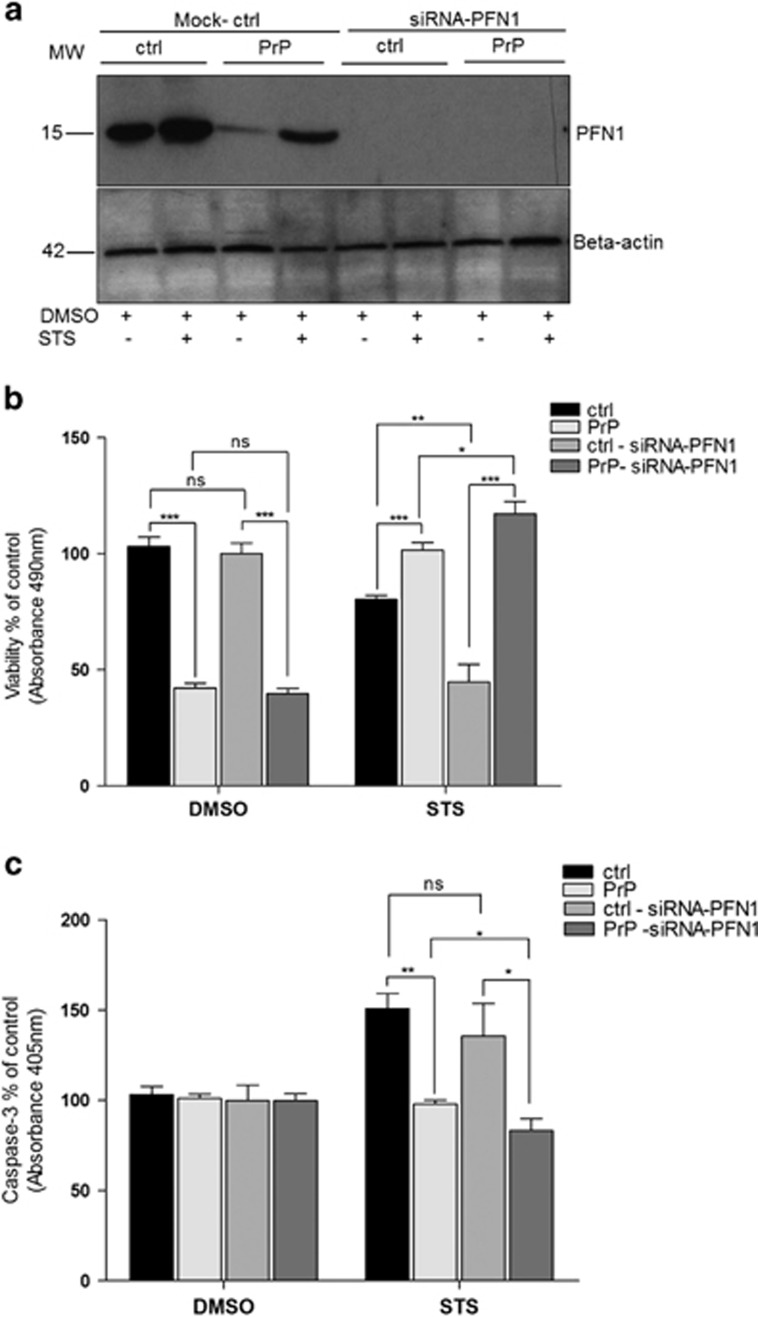
Silencing of profilin-1 expression in SH-SY5Y cells using siRNA. Western blot displays profilin-1 expression after transfection with non-targeting mock control (designated Mock-ctrl) and following transfection with profilin-1 specific siRNA (siRNA-PFN-1). Cells expressing endogenous levels of PrP^c^ (designated ctrl) and PrP^c^-overexpressing cells (designated PrP) were both subjected to 2 h treatment with 1 *μ*M STS or DMSO. Markedly higher profilin-1 expression is present in control *versus* PrP cells both treated with mock control. No profilin-1 expression after transfection with siRNA-PFN-1 was visible after immunoblotting. Beta-actin expression is given as a control for an equal protein load (**a**). Cell viability was measured by MTS assay 24 h post-transfection and 2 h after 1 *μ*M STS or DMSO treatment. Note, increase in cell viability of PrP cells after silencing profilin-1 expression under STS treatment *versus* control cells subjected to the same conditions. PrP cells treated with siRNA-PFN-1 exhibit a significant increase in viability as compared with PrP cells treated with mock control. (**b**) Caspase-3 activity was reduced in PrP cells depleted of profilin-1 and treated with STS as compared with control cells under same experimental conditions. In addition, slight but significant decrease in caspase-3 activity was observed in PrP-siRNA-PFN-1 treated cells as compared with PrP^c^-overexpressing cells not depleted of profilin-1 (PrP), both exposed to STS (**c**). Values represent the mean±S.D. of four independent experiments. Level of significance was calculated using Student's *t*-test: *P⩽0.05; **P⩽0.01; ***P⩽0.001

To test if farther downregulation of PFN-1 has an effect on cell viability and caspase-3 activity of PrP *versus* ctrl cells treated with STS, we performed PFN-1 gene expression silencing using siRNA approach. Western blot analysis demonstrated comparable PFN-1 protein expression pattern after transfection with non-targeting mock control as obtained previously for siRNA-untreated cells (comparison between Figures 8a and 6a). We also verified that level of PrP^c^-overexpression following treatment with siRNA remained unchanged (data not shown). In cells transfected with siRNA-PFN-1, no protein bands neither in ctrl nor in PrP cells were visible (Figure 8a). Cell viability of PrP cells treated with siRNA-PFN-1 *versus* ctrl cells treated with siRNA-PFN-1 both exposed to STS was significantly increased (*P*=0.0006), whereas caspase-3 activity was decreased (*P*=0.0216). In addition, PrP cells treated with siRNA-PFN-1 exhibited significantly higher survival rates (*P*=0.0139) and slightly decreased caspase-3 activity (*P*=0.0311) *versus* PrP cells treated with mock control, both subjected to STS (Figure 8b and c). Remarkably, ctrl cells treated with siRNA-PFN-1 demonstrated lower survival rates (*P*=0.0022) as compared with ctrl cells treated with mock control, both subjected to STS (Figure 8b).

## Discussion

Former reports on PrP^c^ overexpression in different experimental models remain controversial. Wild-type PrP^c^-overexpression can either exert protective effects as shown in BAX and TNF-*α*-mediated cell death *in vitro*^28, 29^ or can induce neurodegeneration and primary myopathy *in vivo*.^14, 16^ Several earlier studies investigating pro- and anti-apoptotic features of PrP^c^, using STS as an apoptotic agent, found that PrP^c^-overexpression renders cells more susceptible to apoptotic cell death, whereas cells devoid of PrP^c^ expression demonstrate reduced susceptibility to STS-induced apoptosis. The mechanism of STS-induced PrP^c^-regulated cell death appears to be p53-dependent and to involve capase-3 activation.^12, 13, 26^ However, the possibility that mechanism of action vary dependent on different cell types and STS concentrations used still persists.^30^ Hence, findings on mouse neuroblastoma N2a cell line showed that downregulation of PrP^c^ sensitizes cells to STS-induced cytotoxicity and apoptosis, whereas an overexpression of PrP^c^ has little or no effect.^18^ Interestingly, in the present study cell viability of PrP^c^-overexpressing cells under both control (cell medium) and DMSO conditions was significantly diminished as compared with SH-SY5Y cells expressing endogenous levels of PrP^c^. However, after exposure to stress conditions (STS treatment) PrP^c^-overexpression seemed to become advantageous and cell survival rates were even higher than in the control group.

The study target was to display early apoptotic proteome and phospho-proteome changes. The functional roles of identified proteins indicate that most STS-provoked apoptotic changes are linked to protein metabolism/folding, energy metabolism, stress response and cytoskeleton organization.

Protein metabolism/folding functional group exhibited the most protein expression/phosphorylation changes following PrP^c^-overexpression in SH-SY5Y cells under employed experimental conditions. For instance, endoplasmic reticulum (ER) stress-activated protein, ER-resident protein 29, a chaperone that facilitates protein processing and transport,^31^ and appears to possess anti-apoptotic properties,^32^ demonstrated an increased expression in PrP^c^-overexpressing *versus* control cells (both treated with STS). In earlier proteomic study, STS treatment of SH-SY5Y cells induced significant changes in endoplasmic reticulum proteins.^33^

A glycolytic enzyme, alpha-enolase, which belongs to energy metabolism functional group of proteins, demonstrated a 5-fold increase in its expression following STS treatment in PrP^c^-overexpressing as compared with control cells underlining the ability of PrP^c^ to attune energy metabolism according to the physiological demands. The expression of this enzyme was clearly enhanced in a study investigating proteome changes in N2a cells after infection with 22 L prion strain.^34^

A cytoskeleton protein that showed significant changes between control and PrP^c^-overexpressing cells after STS treatment was profilin-1 (PFN-1). This protein is ubiquitously expressed actin-binding protein, which when overexpressed facilitates STS-induced apoptosis.^27^ It is well-established that actin-binding proteins are of utmost importance for the adhesion of cells to extracellular matrices (ECM) and for cell survival because of their role in linkage of integrins to actin cytoskeleton.^35^ The fact that apoptotic cells display an early detachment from ECM and the rearrangements of actin cytoskeleton,^36^ an elevated expression of PFN-1 in control as compared with PrP^c^-overexpressing SH-SY5Y cells, both subjected to STS, might indicate an increased susceptibility of the former group to STS-mediated apoptotic cell death. In addition, control *versus* PrP^c^-overexpressing SH-SY5Y cells subjected to DMSO showed likewise significantly higher expression of PFN-1. It appears that STS treatment itself is sufficient to up-regulate PFN-1 expression, whereas PrP^c^ overexpression seems to be able to decrease it. Of interest, N-terminal sequence of PrP^c^ possesses extended poly-(L proline) II (PPII) helix^37^ and profilin family is known to bind to polyproline binding sites.^38^ Profilin-1 interactions with actin and polyproline ligands advance actin polymerization and cell motility,^39^ which becomes important during tumor progression. Hence, downregulation of PFN-1 was reported in different adenocarcinoma (i.e., breast, pancreatic).^40, 41^ Loss of PFN-1 expression led to increased motility and invasiveness of MDA-MB-231 breast cancer cells, whereas PFN-1 overexpression suppressed micro-metastasis of MDA-MB-231 cells in nude mice.^42^ Conversely, an upregulation of PrP^c^ expression was detected in metastatic gastric cancer,^6^ and its expression in pancreatic ductal adenocarcinoma was correlated with a marked decrease in patients' survival.^43^ Interestingly, we showed a significant re-distribution of PFN-1 in PrP^c^-overexpressing *versus* control cells both exposed to STS. Furthermore, silencing of PFN-1 by siRNA resulted in a significant increase in viability and decrease in caspase-3 activity in PrP^c^-overexpressing *versus* control cells under STS conditions. Even more important, a significant raise in viability and concomitant diminishment of caspase-3 activity was observed in PrP^c-^overexpressing cells in which PFN-1 was silenced *versus* PrP^c^-overexpressing cells treated with mock control, both exposed to STS. These findings suggest that downregulation of PFN-1 may have a role in PrP^c^-mediated protection against apoptotic cell death induced by STS. The established link between PFN-1 and PrP^c^ definitely deserves further attention.

Remarkably, p53 another tumor suppressor protein crucial for maintaining genetic stability and cellular redox status^44, 45^ exhibited in the present study nearly identical expression pattern as the one observed for PFN-1. We detected significantly higher levels of p53 expression in control as compared with PrP^c^-overexpressing cells under both experimental conditions. In resting cells p53 levels are low, whereas its levels increase because of rapid stabilization following exposure to different DNA-damaging agents and other stress stimuli.^46^ In earlier study, we observed no differences in p53 expression after stable PrP^c^-overexpression in SH-SY5Y cells.^17^ However, it must be emphasized that in the latter study SH-SY5Y control cells were not stably transfected with an empty pCIneo vector. Nevertheless, p53-dependent activation of caspase-3 was repeatedly verified following STS treatment of both PrP^c^-overexpressing and cells with endogenous PrP^c^ levels.^13, 26^ We suggest that higher expression of PFN-1 and p53 in control as compared with PrP^c^-overexpressing SH-SY5Y cells subjected to STS might contribute to apoptosis (caspase-3 activation) in the former *versus* the latter group.

Transgelin is another protein affecting dynamics of the actin cytoskeleton.^47^ In the present study, western blot analysis exhibited approximately 45-fold higher expression of transgelin-2 in PrP^c^-overexpressing cells subjected to STS as compared with all other experimental conditions. Meanwhile, transgelin was identified as a repressor of metallo-matrix proteinase 9 (MMP-9) expression involved in ECM remodeling.^48, 49^ Likewise, PrP^c^ was shown to decrease MMP-9 transcript levels in neuronal cells.^50^ Moreover, a surplus of MMP-9 pro-apoptotic *versus* anti-apoptotic properties,^51^ especially during neuronal apoptosis, let us assume that PrP^c^-mediated upregulation of transgelin-2 following STS treatment presumably led to downregulation of MMP-9 and, thus to a reduction of its pro-apoptotic properties.

Peroxiredoxin-4 (Prx-4), which belongs to a stress response functional group of proteins is an ER-localized enzyme^52^ that exhibits antioxidant properties and promotes cell survival.^53, 54^ Peroxiredoxin-4 appears to provide a cytoprotective effect against environmental factors that raise levels of hydrogen peroxide in the lumen of ER.^54^ We observed 10-fold higher expression of Prx-4 in PrP^c^-overexpressing *versus* control cells, both subjected to STS. We presume that PrP^c^-overexpressing SH-SY5Y cells subjected to STS undergo less ER stress than the control cells because of their high Prx-4 expression. In line with this connotation, Cusinato *et al.*^55^ showed that when cellular ER stress is present STS effects are more potent.

The fact that protein phosphorylation is the most common post-translational modification in eukaryotic cells involved in all basic cellular processes prompted us to investigate phospho-proteome of PrP^c^-overexpressing SH-SY5Y cells following STS treatment. Inositol monophosphatase 1 (IMPA1) phosphorylation status was decreased in PrP^c^-overexpressing cells subjected to STS as compared with control cells subjected to the same conditions. Inhibition of IMPA1 is characterized by depletion of free inositol and a subsequent decrease of inositol-1,4,5-triphosphate (IP_3_) levels resulting in induction of autophagy,^56^ which is generally known to block the induction of apoptosis.^57^ Another protein's phosphorylation level was exclusively increased by PrP^c^-overexpression treated with STS: calcium-binding mitochondrial carrier protein SCaMC-1. This protein is beneficial for counteracting Ca^2+^ overload-induced cell death,^58^ and STS is known to provoke cytosolic and mitochondrial Ca^2+^ overload, a critical event for initiation of cell death.^59^

Provansal *et al.*^34^ identified two proteins modulated after prion infection, which revealed changed phosphorylation status in the present study: stomatin-like protein 2, involved in mitochondrial biogenesis^60^ and T-complex protein 1, a molecular chaperone. Not only that stomatin-like protein 2 phosphorylation status was decreased in PrP^c^-overexpressing cells, but also its expression level was down-regulated following PrP^c^-overexpression in SH-SY5Y cells treated with STS. On the contrary, T-complex protein 1 showed an increased phosphorylation on PrP^c^ overexpression.

Cystathionine-beta synthase (CBS), an enzyme primarily localized in the brain, important for cellular H_2_S production^61^ exhibited diminished phosphorylation in PrP^c^-overexpressing cells subjected to both DMSO and STS. H_2_S appears to have oxygen sensor properties and to be involved in regulation of energy metabolism.^62^ CBS phosphorylation at Ser 227 has been shown to modulate its catalytic activity and thereby H_2_S production.^63^ The possible role of PrP^c^ in regulating phosphorylation status of CBS remains to be elucidated.

Altogether, PrP^c^-overexpression in SH-SY5Y cells *per se* appears to stress the cells as observed by decreased cell viability under control conditions. However, after exposure to STS, higher levels of PrP^c^ become beneficial in terms of cell survival and apoptosis as compared with endogenous PrP^c^ levels. Proteome and phospho-proteome indicate major changes in protein metabolism/folding, energy metabolism, cytoskeleton and stress response group between the both experimental groups. Although not identified during proteome analysis, we confirm differential regulation of tumor suppressor p53 by different PrP^c^ levels under non-apoptotic and apoptotic conditions.

In our opinion the established link between PrP^c^ and PFN-1 deserves a special attention given PFN-1 role in tumor suppression on one side and PrP^c^ role as a promotor of cancer cell invasiveness on the other. Reduction of PFN-1 expression by elevated PrP^c^ levels might prove important not only as so far unrecognized contributing factor for increased resistance of PrP^c^-overexpressing SH-SY5Y cells to STS-induced apoptosis, but also in the light of emerging roles of these two proteins in cancer.

## Materials and methods

### Cell culture, stable transfection and staurosporine treatment

SH-SY5Y human neuroblastoma cells constitutively and stably expressing full-length human PrP^c^ were established and maintained as previously described.^17^ In addition, control transfected SH-SY5Y cells stably expressing pCIneo plasmid (Promega, Mannheim, Germany) without a human prion protein gene (*PRNP*) insert were generated in the same manner. Both cell lines were grown in Dulbecco's modified Eagle medium (DMEM; Biochrom, Berlin, Germany) supplemented with 10% heat-inactivated fetal calf serum (FCS; Biochrom), 1% penicillin/streptomycin (P/S; Biochrom) and 1% l-glutamine (Biochrom) at 37 °C, 5% CO_2_ supply and 95% humidity. Six weeks after addition of 400 *μ*g/ml Geneticin (Gibco/Invitrogen, Karlsruhe, Germany) both stably transfected cell lines were selected and further maintained in the same medium with a lower Geneticin concentration (200 *μ*g/ml). Before induction of apoptotic cell death by STS in empty vector- and *PRNP*-transfected SH-SY5Y cells, cell medium containing Geneticin was aspirated and a fresh medium without Geneticin was added. Apoptosis was induced by exposure of cells to 1*μ*M STS (Sigma-Aldrich, Taufkirchen, Germany) for 2 h. Staurosporine was dissolved in dimethyl sulfoxide (DMSO; Sigma-Aldrich). Control cells received vehicle, 0.1% DMSO. After 2 h of exposure to either STS or DMSO cells were washed twice with phosphate-buffered saline (PBS) and collected for further analyses.

### siRNA and transfection

For silencing of profilin-1 gene expression 200 000 cells per well in a 6-well plate were seeded in complete DMEM medium (Biochrom) before transient transfection with siRNA duplexes, using Lipofectamine 2000 (Invitrogen, Carlsbad, CA, USA) according to the manufacturer's instructions. After 24 h in complete DMEM, cells were re-suspended in Opti-MEM I reduced serum medium (ThermoFischer Scientific, Darmstadt, Germany) and transfected either with 200 pmol/l profilin-1-specific siRNAs (catalog number sc-36316-Santa Cruz Biotechnology, Dallas, TX, USA) containing three different sequences: GUGUCCUGGUUGGCAAAGA, CACGGUGGUUUGAUCAACA, and CCCCAUACCCCUUAUUGCU^64^ or with equimolar non-targeting siRNA duplex (control siRNA duplex negative control: Eurogentec, Cologne, Germany) as a negative control. Six hours post-transfection Opti-MEM was replaced with complete DMEM and cells were incubated for another 18 h. Subsequently, 1*μ*M STS or DMSO were added for another 2 h before processing cells for MTS proliferation and caspase-3 activity assay (please see below).

### Silver staining and densitometric analysis

The 17cm gels were silver stained as previously described.^65^ Briefly, gels were fixed in 50% methanol, 12% acetic acid for 1 h at room temperature (RT) on an orbital shaker, washed for 40 min in 50% ethanol and sensitized with 0.8 mM sodium thiosulfate for 60 s. The gels were then incubated in freshly prepared silver nitrate solution (0.2% silver nitrate and 0.026% formaldehyde) for 20 min at RT followed by three washing steps of 20 s each, in distilled water. Finally, gels were placed in a developing solution (6% sodium carbonate, 0.0185% formaldehyde and 0.05% sodium thiosulfate) until standard markers were stained completely and adequate spots visualized. Gels were scanned with a Gel CanoScan 8400 F (Canon, Tokyo, Japan). Densitometric analyses were performed using Delta 2D software version 3.6 (Decodon GmbH, Greifswald, Germany). Furthermore, the intensities of the spots were normalized by dividing the intensity of each spot by the sum of all spot intensities on the corresponding gels. Fold change, standard deviation and Student's *t*-test significance level were calculated using Microsoft excel software. Spots having at least 1.5-fold expressional changes (*P*⩽0.05) were considered statistically significant. The spots of interest were excised from the silver stained gels, in-gel protein digested and extracted peptides were subjected to the mass spectrometric based MS/MS protein identification analysis (please see Supplementary Table 1) as described earlier.^66^ Acceptance criteria was set to at least two sequenced peptides per protein spot. Four independent 2-DE experiments were performed.

### Phospho-specific staining of 2-DE gels

Gels were fixed twice in a solution containing 50% methanol and 10% acetic acid for 45 min and washed three times in double distilled water for 15 min each. Gels were incubated in Pro-Q Diamond phospho-stain (Invitrogen, Paisley, UK) overnight in the dark at RT, distained in 20% acetonitrile and 50 mM sodium acetate three times for 30 min, followed by three washing steps in double distilled water for 5 min each. Gels were scanned using an imaging instrument (FLA -5100 Fuji photo film, Dusseldorf, Germany) at a wavelength of 532 nm. Fold change, standard deviation and Student's *t*-test level of significance were calculated using Microsoft excel software. Spots having at least 1.4-fold expressional changes (*P*⩽0.05) were considered statistically significant. In-gel protein digestion and MS/MS analysis (please see Supplementary Table 2) were performed as described in the section ‘Silver staining and densitometric analysis'. Acceptance criteria was set to at least two sequenced peptides per protein spot. Four independent 2-DE experiments were performed.

### Antibodies and immunoblot analysis

Primary antibodies used for immunoblot analysis were monoclonal mouse anti-transgelin-2 (1:1000) (Abcam, Cambridge, UK), monoclonal mouse anti-p53 (1:1000) (Abcam), monoclonal mouse anti-beta-actin (1:5000 Abcam) and monoclonal rabbit anti-profilin-1 (1:1000) (Abcam). Secondary antibodies used were horseradish peroxidase (HRP)-conjugated rabbit anti-mouse (IBA, Goettingen, Germany) and goat anti-rabbit (Jackson Immunoresearch Laboratories, Palo Alto, CA, USA).

Cell lysis extraction and immunoblotting were performed as described previously.^67^ Briefly, cells were lysed (50 mM Tris–HCl, pH=8, 1% Triton X-100, 0.5% CHAPS, 1 mM DTT), and lysates were cleared of cell debris (1 min at 1000 × *g*, 4 °C). Cell lysates were supplemented with protease and phosphatase inhibitor cocktail (Roche, Mannheim, Germany) and were separated on 12.5% SDS-PAGE. Immunoblotting was performed using above mentioned primary antibodies overnight at 4 °C. Membranes were then rinsed three times in 1 × Tris-buffered saline with Tween-20 (TBS-T) and incubated with the corresponding HRP-conjugated secondary antibody (diluted 1:2000; 1:5000) for 1 h at RT. Immunoreactivity was detected after immersion of the membranes into enhanced chemiluminescence (ECL) solution and exposure to ECL-Hyperfilm (Amersham Biosciences, Buckinghamshire, UK). Images were documented using the ScanMaker4 (Microtek, Kiel, Germany, International), after correction for the background, and band intensities were determined by densitometry using Labimage (version 2.7.1, Kapelan GmbH, Germany) data analyzer software. For each condition analysed, three western blots were prepared from three or four different protein extractions.

### Determination of cell viability

Cell viability was determined by the MTS proliferation assay (Promega, Madison, WI, USA), which measures reduction of [3-(4,5–dimethylthiazol–2–yl)-5-(3-carboxymethoxyphenyl)-2-(-4-sulfophenyl)-2H-tetrazolium] (MTS) to a water soluble formazan salt, which occurs only in metabolically active cells. Both control- and *PRNP*-stably transfected SH-SY5Y neuroblastoma cells were grown in 75 cm^2^ culture flasks at 37 °C in a 5% CO_2_ atmosphere until reaching 60–70% confluency. Subsequently, cells were either treated with 1 *μ*M STS or DMSO (vehicle control) for 2 h or were left untreated (negative control), rinsed with cold 1 × PBS and detached from the flask surface using trypsin-EDTA solution. After centrifugation at 400 × *g* for 5 min at 4 °C cells were re-suspended in a cell culture medium, seeded into 24-well plates (Nunc, Roskilde, Denmark) at cell density of 1 × 10^5^ cells per well and incubated for 12 h at 37 °C. The cell viability was quantitatively assessed by using MTS reagent in the presence of phenazine methyl sulphate (PMS). After addition of combined MTS/PMS solution into each well, plates were incubated in a humidified atmosphere containing 5% CO_2_ for 1 h at 37 °C. The absorbance values were read at 490 nm using a Multiscan plate reader (Labsystems, Manassas, VA, USA) and Accent software 2.6. Cell-free background absorbance of the medium incubated with the MTS reagent was subtracted from sample wells to obtain the final absorbance values. All MTS assays were performed in triplicates.

### Caspase-3 activity measurements

The caspase-3 activity assay (Promega) enables quantitative assessment of caspase-3 (DEVDase) protease activity, an early apoptotic event. The assay was performed according to the manufacturer's recommendations. Briefly, the cell density of empty vector- and *PRNP*-stably transfected SH-SY5Y cells was adjusted to 5 × 10^5^ cells per ml. Twenty-four hours later cells were either left untreated (negative control), were treated with DMSO (vehicle control) or with 1 *μ*M STS for 2 h at 37 °C in a humidified, 5% CO_2_ atmosphere. As an additional control for inhibition of apoptosis, the caspase inhibitor Z-VAD-FMK was concomitantly added in a final concentration of 50 *μ*M to a fraction of cells treated with 1*μ*M STS. Afterwards, cells were rinsed with ice-cold PBS, gently scraped, collected by centrifugation and finally lysed in the cell lysis buffer for 15 min at 4 °C. Cell lysates were centrifuged at 15 000 × *g* for 20 min at 4 °C. Protein concentration was estimated by the Bradford assay (Bio-Rad, Munich, Germany) and the total cell lysate (50 *μ*g) was then incubated with 200 *μ*M caspase-3 specific substrate DEVD-pNA for 4–5 h at 37 °C. Caspase-3-mediated release of *p*-nitroaniline (pNA) was measured by absorbance at 405 nm. Background absorbance from the control (untreated cells) was subtracted from the samples after the final absorbance was obtained. All caspase assays were performed in quadruplicates.

### PrP^c^ ELISA

PrP^c^ ELISA was performed by using BSE-Detection Kit (Bio-Rad Laboratories GmbH) according to the instructions of the supplier. Briefly, equal amounts of protein from empty vector- and *PRNP*-stably transfected SH-SY5Y cells were added to a microtiter plate coated with a monoclonal anti-PrP antibody and incubated for 75 min at 37 °C. Recombinant human prion protein (Prionics, Zurich, Switzerland) was used as a positive control. After several washing steps a monoclonal anti-PrP antibody was added followed by incubation for 60 min at 4 °C. After incubation with a developing solution containing H_2_O_2_ and tetramethylbenzidine (30 min at RT) reaction was stopped, extinction was measured (at 450/620 nm) and PrP^c^ content was determined (ng/*μ*l protein).

### Statistical analysis

Densitometric values of 2-DE gels and western blot bands as well as the values obtained from cell viability and caspase-3 activity assays were statistically assessed using unpaired two-sided Student's *t*-test. Means and S.D. were calculated from three to four independent set of experiments. Differences with *P*⩽0.05 value were considered statistically significant.

## Figures and Tables

**Table 1:** List of proteins identified from 2-DE gels of control and PrP^c^-overexpressing SH-SH5Y cells treated either with DMSO or STS

*Groups compared*	*Spot no.*	*Fold change*	*Peptides matched*	*Score*	*State change*	P-*value*	*Protein ID*	*Accession no.*
PrP^+STS^/ctrl^+STS^	375	1.95	10	166	↑ PrP^+STS^	0.003	Succinate dehydrogenase (ubiquinone) flavoprotein subunit, mitochondrial	P31040
	384	1.60	9	127	↑ PrP^+STS^	0.048	Actin-interacting protein 1	O75083
	607	5.06	15	523	↑ PrP^+STS^	0.021	Alpha-enolase	P06733
	702	1.52	3	81	↓ PrP^+STS^	0.049	Stomatin-like protein 2	Q9UJZ1
	1006	1.95	11	205	↓ PrP^+STS^	0.011	Proteasome subunit alpha type-3	P25788
	1049	3.19	3	100	↓ PrP^+STS^	0.020	Alpha N-terminal protein methyltransferase 1 A	Q9BV86
	1042	10.16	6	92	↑ PrP^+STS^	0.0008	Peroxiredoxin-4	Q13162
	1016	1.60	14	395	↑ PrP^+STS^	0.011	Endoplasmic reticulum resident protein 29	P30040
	1105	1.76	5	54	↓ PrP^+STS^	0.018	Ubiquitin-conjugating enzyme E2 O	Q9C0C9
	1161	1.54	8	124	↓ PrP^+STS^	0.048	Adenine phosphoribosyl transferase	P07741
	1157	3.65	22	393	↑ PrP^+STS^	0.008	Transgelin-2	P37802
	1293	2.07	7	136	↓ PrP^+STS^	0.032	40 S ribosomal protein S12	P25398
	1294	1.64	32	627	↓ PrP^+STS^	0.042	Profilin-1	P07737
PrP^+DMSO^/ ctrl^+DMSO^	1108	2.45	3	86	↑ PrP^+DMSO^	0.019	Ubiquitin-conjugating enzyme E2 K	Q9Y2D3
	1294	1.64	6	263	↑ PrP^+DMSO^	0.042	Profilin-1	P07737
	1161	1.84	8	124	↓ PrP^+DMSO^	0.011	Adenine phosphoribosyl transferase	P07741
PrP^+DMSO^/ctrl^+STS^	—	—	—	—	—	—	—	—
ctrl^+STS^/ctrl^+DMSO^	375	1.59	10	166	↓ ctrl^+STS^	0.020	Succinate dehydro -genase (ubiquinone) flavoprotein subunit, mitochondrial	P31040
	1108	1.90	3	86	↑ ctrl^+STS^	0.028	Ubiquitin-conjugating enzyme E2 K	Q9Y2D3
	1161	1.58	8	124	↓ ctrl^+STS^	0.009	Adenine phosphoribosyl transferase	P07741
	1294	2.07	6	263	↑ ctrl^+STS^	0.013	Profilin-1	P07737
PrP^+STS^/PrP^+DMSO^	384	1.90	9	127	↑ PrP^+STS^	0.024	Actin-interacting protein 1	O75083
PrP^+STS^/ctrl^+DMSO^	—	—	—	—	—	—	—	—

Fourteen different proteins were identified from 2-DE gels of SH-SY5Y cells stably transfected either with pCIneo*PRNP* or an empty vector. The number of spots corresponds to their location on the gel (Figure 3). The comparison of protein regulation between distinct transfection groups, fold change, number of peptides matched, ion score, state change, significance (unpaired Student's *t*-test), protein identification and Swiss-Prot accession numbers are given for each spot. The depiction of transfection groups is as follows: ctrl^+DMSO^=empty vector transfected cells treated with DMSO; ctrl^+STS^=empty vector transfected cells treated with STS; PrP^+DMSO^=PrP^c^-overexpressing cells treated with DMSO; PrP^+STS^=PrP^c^-overexpressing cells treated with STS

**Table 2:** List of phosphorylated proteins identified from 2-DE gels of control and PrPc-overexpressing SH-SH5Y cells treated either with DMSO or STS

*Groups compared*	*Spot no.*	*Fold change*	*Peptides matched*	*Score*	*State change*	P-*value*	*Protein ID*	*Accession no.*
ctrl^+STS^/PrP^+STS^	829	0.66	2	50	↓ PrP^+STS^	0.031	Inositol monophosphatase 1	P29218
	517	1.45	3	85	↑ PrP^+STS^	0.029	Calcium-binding mitochondrial carrier protein SCaMC-1	Q6NUK1
ctrl^+DMSO^/ PrP^+DMSO^	646	0.65	15	210	↓ PrP^+DMSO^	0.047	Stomatin-like protein 2	Q9UJZ1
	390	0.68	6	50	↓ PrP^+DMSO^	0.033	Cystathionine-beta-synthase	P35520
	355	1.49	5	21	↑ PrP^+DMSO^	0.041	T-complex protein 1	P49368
								
ctrl^+STS^/ PrP^+DMSO^	1175	1.45	8	112	↑ PrP^+DMSO^	0.044	Rho GTPase-activating protein 1	Q07960
ctrl^+DMSO^/ctrl^+STS^	651	0.65	4	80	↓ ctrl^+STS^	0.014	26 S protesome non-ATPase regulatory subunit 13	Q9UNM6
PrP^+DMSO^/PrP^+STS^	390	0.68	6	50	↓ PrP^+STS^	0.0075	Cystathionine-beta-synthase	P35520
ctrl^+DMSO^/ PrP^+STS^	394	0.64	—	—	↓ PrP^+STS^	0.043	Unidentified	NA
	595	0.67	25	274	↓ PrP^+STS^	0.046	Eukaryotic translation initiation factor 3 subunit 1	Q13347
	474	1.44	9	151	↑ PrP^+STS^	0.037	40 kDa peptidyl-prolyl-cis-trans isomerase	Q08752

Ten different proteins were identified from phospho-stained 2-DE gels of SH-SY5Y cells stably transfected either with pCIneo*PRNP* or an empty vector (protein spot no. 394 remained unidentified). The number of spots corresponds to their location on the gel (Figure 4). The comparison of protein regulation between distinct transfection groups, fold change, number of peptides matched, ion score, state change, significance (unpaired Student's *t*-test), protein identification and Swiss-Prot accession numbers are given for each spot. The depiction of transfection groups is as given in Table 1
