# Supplementary material for: Cellular prion protein mediates early apoptotic proteome alternation and phospho-modification in human neuroblastoma cells
Source: Cell Death Dis. 2017 Jan 19;8(1):e2557–. doi: 10.1038/cddis.2016.384 (PMC5386350; doi:10.1038/cddis.2016.384)
Supplement: Supplementary Figure 1 [file cddis2016384x1.pdf]

| Groups compared | Spot no. | State change | Protein ID |
|---|---|---|---|
| PrP$^{+STS}$/ ctrl$^{+STS}$ | 375 | ↑ PrP$^{+STS}$ | Succinate dehydro - genase (ubiquinone) flavoprotein subunit, mitochondrial |

```
  1 MSGVRGLSRL LSARRLALAK AWPTVLQTGT RGFHFTVDGN KRASAKVSDS
 51 ISAQYPVVDH EFDAVVVGAG GAGLRAAFGL SEAGFNTACV TKLFPTRSHT
101 VAAQGGINAA LGNMEEDNWR WHFYDTVKGS DWLGDQDAIH YMTEQAPAAV
151 VELENYGMPF SRTEDGKIYQ RAFGGQSLKF GKGGQAHRCC CVADRTGHSL
201 LHTLYGRSLR YDTSYFVEYF ALDLLMENGE CRGVIALCIE DGSIHRIRAK
251 NTVVATGGYG RTYFSCTSAH TSTGDGTAMI TRAGLPCQDL EFVQFHPTGI
301 YGAGCLITEG CRGEGGILIN SQGERFMERY APVAKDLASR DVVSRSMTLE
351 IREGRGCGPE KDHVYLQLHH LPPEQLATRL PGISETAMIF AGVDVTKEPI
401 PVLPTVHYNM GGIPTNYKGQ VLRHVNGQDQ IVPGLYACGE AACASVHGAN
451 RLGANSLLDL VVFGRACALS IEESCRPGDK VPPIKPNAGE ESVMNLDKLR
501 FADGSIRTSE LRLSMQKSMQ NHAAVFRVGS VLQEGCGKIS KLYGDLKHLK
551 TFDRGMVWNT DLVETLELQN LMLCALQTIY GAEARKESRG AHAREDYKVR
601 IDEYDYSKPI QGQQKKPFEE HWRKHTLSYV DVGTGKVTLE YRPVIDKTLN
651 EADCATVPPA IRSY
```

| Query | Start - End | Observed | Mr(expt) | Mr(calc) | Delta | M | Score | Expect | Rank | U | Peptide |
|---|---|---|---|---|---|---|---|---|---|---|---|
| 147 | 76 - 92 | 872.4245 | 1742.8344 | 1742.8298 | 0.0047 | 0 | 29 | 0.89 | 1 | U | R.AAFGLSEAGFNTACVTK.L |
| 61 | 251 - 261 | 547.7744 | 1093.5342 | 1093.5516 | -0.0174 | 0 | 12 | 51 | 2 | U | K.NTVVATGGYGR.T |
| 111 | 313 - 325 | 665.3373 | 1328.6600 | 1328.6684 | -0.0084 | 0 | 38 | 0.14 | 1 | U | R.GEGGILINSQGER.F |
| 112 | 313 - 325 | 665.3375 | 1328.6604 | 1328.6684 | -0.0080 | 0 | 32 | 0.55 | 1 | U | R.GEGGILINSQGER.F |
| 129 | 452 - 465 | 737.4243 | 1472.8340 | 1472.8351 | -0.0011 | 0 | 112 | 5.6e-09 | 1 | U | R.LGANSLLDLVVFGR.A |
| 131 | 452 - 465 | 737.4254 | 1472.8362 | 1472.8351 | 0.0011 | 0 | 7 | 1.7e+02 | 1 | U | R.LGANSLLDLVVFGR.A |
| 95 | 518 - 527 | 588.7869 | 1175.5592 | 1175.5506 | 0.0087 | 0 | 4 | 3.9e+02 | 2 | U | K.SMQNHAAVFR.V + Oxidation (M) |
| 106 | 625 - 636 | 638.8265 | 1275.6384 | 1275.6459 | -0.0075 | 0 | 2 | 6e+02 | 1 | U | K.HTLSYVDVGTGK.V |
| 136 | 648 - 662 | 814.4075 | 1626.8004 | 1626.8035 | -0.0031 | 0 | 47 | 0.016 | 1 | U | K.TLNEADCATVPPAIR.S |
| 137 | 648 - 662 | 814.4078 | 1626.8010 | 1626.8035 | -0.0025 | 0 | 6 | 2e+02 | 1 | U | K.TLNEADCATVPPAIR.S |

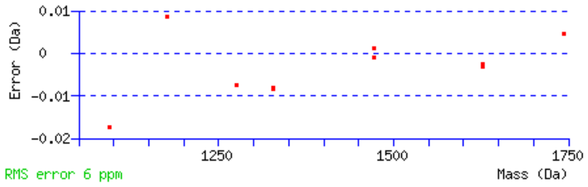


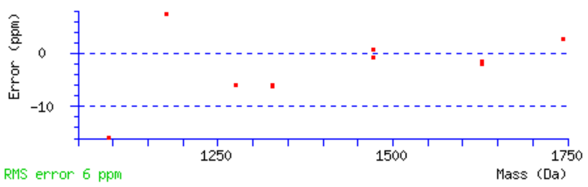

| | | | |
|---|---|---|---|
| 384 | ↑ PrP+STS | Actin-interacting protein 1 | |

```
  1 MPYEIKKVFA SLPQVERGVS KIVGGDPKGN SFLYTNGKCV ILRNIDNPAI
 51 ADIYTEHAHQ VVVAKYAPSG FYIASGDVSG KLRIWDTTQK EHLLKYEYQP
101 FAGKIKDIAW TEDSKRIAVV GEGREKFGAV FLWDSGSSVG EITGHNKVIN
151 SVDIKQSRPY RLVTGSDDNC AAFFEGPPFK FKFTISDHGR FVNCVRFSPD
201 GNRFATASAD GQIFIYDGKT GEKVCALGGS KAHDGGIYAI SWSPDSTHLL
251 SASGDKTSKI WDVNVNSVVN TFTMGSNVLD QQLGCLWQKD HLLSISLSGY
301 INYLDKNNPS KPLRVIKGHS KSIQCLTVHK NGGKSYIYSG SHDGHINYWD
351 SETGENDSFA GKGHTNQVSR MTVDEHGQLV SCSMDDTVRY TNLTLRDYSG
401 QGVVKLDVQP KCLAVGPGGY TVVVCIGQIV LLKDQRKCFS IDNPGYEPEV
451 VAVHPGGETV AVGGADGNVR LYSILGTTLK DEGKLLEAKG PVTDLAFSHD
501 GAFLAVCDAS KVVTVFSVAD GYSENNVFYG HHAKIVCLAW SPDNEHFASG
551 GMDMMVYVWT LSDPETRVKI QDAHRLHHVS SLAWLDEHTL VTTSHDASVK
601 EWTIAY
```

| Query | Start – End | Observed | Mr(expt) | Mr(calc) | Delta | M | Score | Expect | Rank | U | Peptide |
|---|---|---|---|---|---|---|---|---|---|---|---|
| 72 | 8 – 17 | 573.3154 | 1144.6162 | 1144.6241 | -0.0078 | 0 | 51 | 0.0014 | 1 | U | K.VFASLPQVER.G |
| 73 | 8 – 17 | 573.3155 | 1144.6164 | 1144.6241 | -0.0076 | 0 | 67 | 3.1e-05 | 1 | U | K.VFASLPQVER.G |
| 74 | 8 – 17 | 573.3158 | 1144.6170 | 1144.6241 | -0.0070 | 0 | 71 | 1.3e-05 | 1 | U | K.VFASLPQVER.G |
| 65 | 96 – 104 | 551.7612 | 1101.5078 | 1101.5131 | -0.0052 | 0 | 1 | 1.3e+02 | 1 | U | K.YEYQPFAGK.I |
| 55 | 397 – 405 | 476.7359 | 951.4572 | 951.4662 | -0.0089 | 0 | 4 | 62 | 1 | U | R.DYSGQGVVK.L |
| 66 | 471 – 480 | 554.8296 | 1107.6446 | 1107.6539 | -0.0093 | 0 | 2 | 99 | 1 | U | R.LYSILGTTLK.D |
| 67 | 471 – 480 | 554.8297 | 1107.6448 | 1107.6539 | -0.0091 | 0 | 3 | 68 | 1 | U | R.LYSILGTTLK.D |
| 94 | 471 – 484 | 513.2806 | 1536.8200 | 1536.8399 | -0.0199 | 1 | 8 | 20 | 1 | U | R.LYSILGTTLKDEGK.L |
| 95 | 471 – 484 | 513.2807 | 1536.8203 | 1536.8399 | -0.0196 | 1 | 5 | 36 | 1 | U | R.LYSILGTTLKDEGK.L |

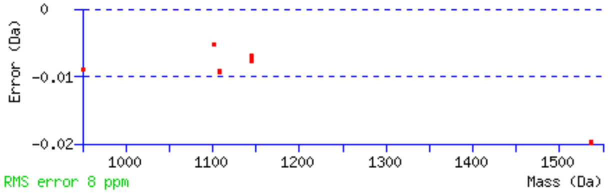


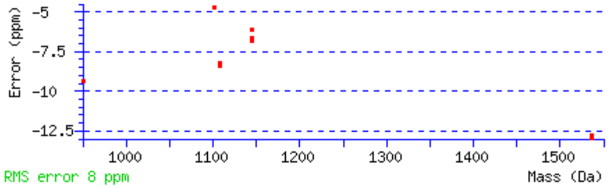

607 ↑ $PrP^{+STS}$ Alpha-enolase

```
  1 MSILKIHARE IFDSRGNPTV EVDLFTSKGL FRAAVPSGAS TGIYEALELR
 51 DNDKTRYMGK GVSKAVEHIN KTIAPALVSK KLNVTEQEKI DKLMIEMDGT
101 ENKSKFGANA ILGVSLAVCK AGAVEKGVPL YRHIADLAGN SEVILPVPAF
151 NVINGGSHAG NKLAMQEFMI LPVGAANFRE AMRIGAEVYH NLKNVIKEKY
201 GKDATNVGDE GGFAPNILEN KEGLELLKTA IGKAGYTDKV VIGMDVAASE
251 FFRSGKYDLD FKSPDDPSRY ISPDQLADLY KSFIKDYPVV SIEDPFDQDD
301 WGAWQKFTAS AGIQVVGDDL TVTNPKRIAK AVNEKSCNCL LLKVNQIGSV
351 TESLQACKLA QANGWGVMVS HRSGETEDTF IADLVVGLCT GQIKTGAPCR
401 SERLAKYNQL LRIEEELGSK AKFAGRNFRN PLAK
```

| Query | Start – End | Observed | Mr(expt) | Mr(calc) | Delta | M | Score | Expect | Rank | U | Peptide |
|---|---|---|---|---|---|---|---|---|---|---|---|
| 72 | 16 – 28 | 703.8607 | 1405.7068 | 1405.7089 | -0.0021 | 0 | 79 | 1.2e-05 | 1 | U | R.GNPTVEVDLFTSK.G |
| 73 | 16 – 28 | 703.8607 | 1405.7068 | 1405.7089 | -0.0021 | 0 | 84 | 3.9e-06 | 1 | U | R.GNPTVEVDLFTSK.G |
| 98 | 33 – 50 | 902.9638 | 1803.9130 | 1803.9366 | -0.0236 | 0 | 125 | 1.9e-10 | 1 | | R.AAVPSGASTGIYEALELR.D |
| 99 | 33 – 50 | 902.9639 | 1803.9132 | 1803.9366 | -0.0234 | 0 | 118 | 9.7e-10 | 1 | | R.AAVPSGASTGIYEALELR.D |
| 100 | 33 – 50 | 902.9639 | 1803.9132 | 1803.9366 | -0.0234 | 0 | 130 | 6.5e-11 | 1 | | R.AAVPSGASTGIYEALELR.D |
| 37 | 72 – 80 | 450.2759 | 898.5372 | 898.5488 | -0.0115 | 0 | 12 | 58 | 1 | | K.TIAPALVSK.K |
| 63 | 81 – 89 | 544.6949 | 1087.3752 | 1087.5873 | -0.2121 | 1 | 38 | 0.16 | 1 | U | K.KLNVTEQEK.I |
| 91 | 240 – 253 | 778.8901 | 1555.7656 | 1555.7705 | -0.0048 | 0 | 1 | 5.8e+02 | 1 | U | K.VVIGMDVAASEFFR.S + Oxidation (M) |
| 93 | 240 – 253 | 778.8904 | 1555.7662 | 1555.7705 | -0.0042 | 0 | 6 | 1.9e+02 | 1 | U | K.VVIGMDVAASEFFR.S + Oxidation (M) |
| 75 | 270 – 281 | 713.3547 | 1424.6948 | 1424.7187 | -0.0239 | 0 | 57 | 0.0017 | 1 | U | R.YISPDQLADLYK.S |
| 76 | 270 – 281 | 713.3547 | 1424.6948 | 1424.7187 | -0.0239 | 0 | 33 | 0.4 | 1 | U | R.YISPDQLADLYK.S |
| 77 | 270 – 281 | 713.3549 | 1424.6952 | 1424.7187 | -0.0235 | 0 | 62 | 0.0005 | 1 | U | R.YISPDQLADLYK.S |
| 52 | 336 – 343 | 504.2519 | 1006.4892 | 1006.4940 | -0.0047 | 0 | 15 | 38 | 2 | | K.SCNCLLLK.V |
| 53 | 336 – 343 | 504.2519 | 1006.4892 | 1006.4940 | -0.0047 | 0 | 31 | 0.83 | 2 | | K.SCNCLLLK.V |
| 54 | 336 – 343 | 504.2521 | 1006.4896 | 1006.4940 | -0.0043 | 0 | 16 | 30 | 2 | | K.SCNCLLLK.V |

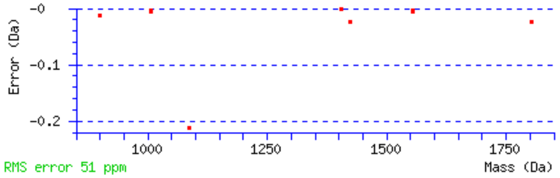


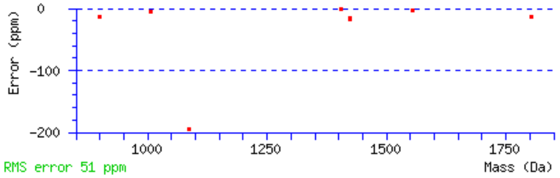

702 ↓ PrP$^{+STS}$ Stomatin-like protein 2

Matched peptides shown in ***bold red***.

1 MLARAARGTG ALLLRGSLLA SGRAPRRASS GLPRNTVVLF VPQQEAWVVE
51 RMGRFHRILE PGLNILIPVL DRIRYVQSLK EIVINVPEQS AVTLDNVTLQ
101 IDGVLYLRIM DPYKASYGVE DPEYAVTQLA QTTMRSELGK **LSLDKVFR**ER
151 ESLNASIVDA INQAADCWGI RCLRYEIKDI HVPPRVKESM QMQVEAERRK
201 RATVLESEGT R**ESAINVAEG** **KK**QAQILASE AEKAEQINQA AGEASAVLAK
251 AKAKAEAIRI LAAALTQHNG DAAASLTVAE QYVSAFSKLA KDSNTILLPS
301 NPGDVTSMVA QAMGVYGALT KAPVPGTPDS LSSGSSR**DVQ** **GTDASLDEEL**
351 **DR**VKMS

| Query | Start - End | Observed | Mr(expt) | Mr(calc) | Delta | M | Score | Expect | Rank | U | Peptide |
|---|---|---|---|---|---|---|---|---|---|---|---|
| 42 | 141 - 148 | 489.2835 | 976.5524 | 976.5706 | -0.0181 | 1 | 7 | 2.1e+02 | 6 | U | K.LSLDKVFR.E |
| 57 | 212 - 222 | 573.3033 | 1144.5920 | 1144.6088 | -0.0167 | 1 | 13 | 50 | 1 | U | R.ESAINVAEGKK.Q |
| 86 | 338 - 352 | 831.8676 | 1661.7206 | 1661.7380 | -0.0174 | 0 | 81 | 5.8e-06 | 1 | U | R.DVQGTDASLDEELDR.V |

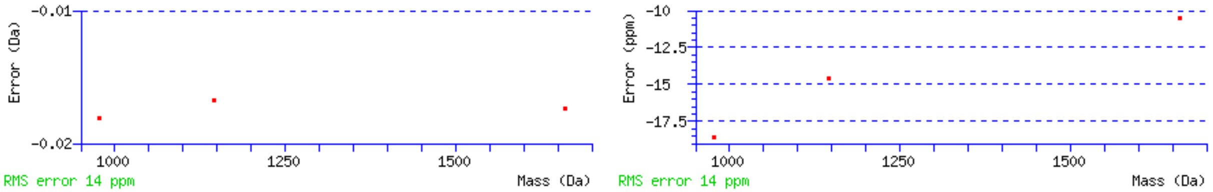


1006 ↓ PrP$^{+STS}$ Proteasome subunit alpha type-3

Matched peptides shown in ***bold red***.

1 MSSIGTGYDL SASTFSPDGR **VFQVEYAMKA** **VENSSTAIGI** **R**CKDGVVFGV
51 EKLVLSK**LYE** **EGSNKR**LFNV DRHVGMAVAG LLADAR**SLAD** **IAREEASNFR**
101 **SNFGYNIPLK** **HLADR**VAMYV HAYTLYSAVR PFGCSFMLGS YSVNDGAQLY
151 MIDPSGVSYG YWGCAIGKAR QAAK**TEIEKL** **QMK**EMTCRDV VKEVAKIIYI
201 VHDEVKDKAF ELELSWVGEI TNGRHEIVPK DVREEAEKYA KESLKEEDES
251 DDDNM

| Query | Start – End | Observed | Mr(expt) | Mr(calc) | Delta | M | Score | Expect | Rank | U | Peptide |
|---|---|---|---|---|---|---|---|---|---|---|---|
| 70 | 21 – 29 | 565.7610 | 1129.5074 | 1129.5478 | -0.0403 | 0 | 35 | 0.3 | 1 | U | R.VFQVEYAMK.A + Oxidation (M) |
| 71 | 21 – 29 | 565.7610 | 1129.5074 | 1129.5478 | -0.0403 | 0 | 31 | 0.8 | 1 | U | R.VFQVEYAMK.A + Oxidation (M) |
| 72 | 21 – 29 | 565.7614 | 1129.5082 | 1129.5478 | -0.0395 | 0 | 29 | 1.2 | 1 | U | R.VFQVEYAMK.A + Oxidation (M) |
| 77 | 30 – 41 | 609.2929 | 1216.5712 | 1216.6411 | -0.0699 | 0 | 70 | 9.2e-05 | 1 | U | K.AVENSSTAIGIR.C |
| 78 | 30 – 41 | 609.2932 | 1216.5718 | 1216.6411 | -0.0693 | 0 | 67 | 0.00021 | 1 | U | K.AVENSSTAIGIR.C |
| 79 | 30 – 41 | 609.3109 | 1216.6072 | 1216.6411 | -0.0339 | 0 | 74 | 4.5e-05 | 1 | U | K.AVENSSTAIGIR.C |
| 68 | 58 – 66 | 548.2570 | 1094.4994 | 1094.5356 | -0.0362 | 1 | 17 | 17 | 1 | U | K.LYEEGSNKR.L |
| 138 | 87 – 100 | 526.9103 | 1577.7091 | 1577.7797 | -0.0707 | 1 | 58 | 0.0011 | 1 | U | R.SLADIAREEASNFR.S |
| 139 | 87 – 100 | 526.9103 | 1577.7091 | 1577.7797 | -0.0707 | 1 | 15 | 24 | 1 | U | R.SLADIAREEASNFR.S |
| 145 | 101 – 115 | 582.2868 | 1743.8386 | 1743.9056 | -0.0671 | 1 | 5 | 2.5e+02 | 1 | U | R.SNFGYNIPLKHLADR.V |
| 73 | 175 – 183 | 568.2835 | 1134.5524 | 1134.5954 | -0.0430 | 1 | 6 | 2.7e+02 | 1 | U | K.TEIEKLQMK.E + Oxidation (M) |

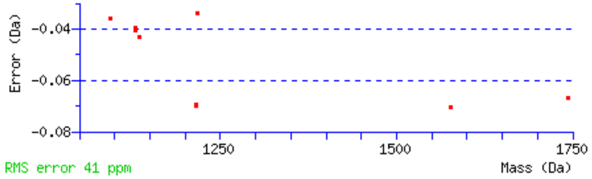


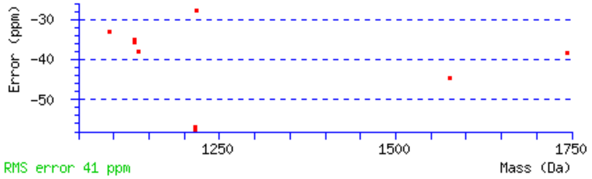


1049 ↓ PrP+STS Alpha N-terminal protein methyltransferase 1A

```
  1 MTSEVIEDEK QFYSKAKTYW KQIPPTVDGM LGGYGHISSI DINSSRKFLQ
 51 RFLREGPNKT GTSCALDCGA GIGRITKRLL LPLFREVDMV DITEDFLVQA
101 KTYLGEEGKR VRNYFCCGLQ DFTPEPDSYD VIWIQWVIGH LTDQHLAEFL
151 RRCKGSLRPN GIIVIKDNMA QEGVILDDVD SSVCRDLDVV RRIICSAGLS
201 LLAEERQENL PDEIYHVYSF ALR
```

| Query | Start – End | Observed | Mr(expt) | Mr(calc) | Delta | M | Score | Expect | Rank | U | Peptide |
|---|---|---|---|---|---|---|---|---|---|---|---|
| 11 | 79 – 85 | 436.2770 | 870.5394 | 870.5691 | -0.0296 | 0 | 15 | 36 | 2 | U | R.LLLPLFR.E |
| 46 | 193 – 206 | 766.3939 | 1530.7732 | 1530.8075 | -0.0343 | 0 | 100 | 8.9e-08 | 1 | U | R.IICSAGLSLLAEER.Q |

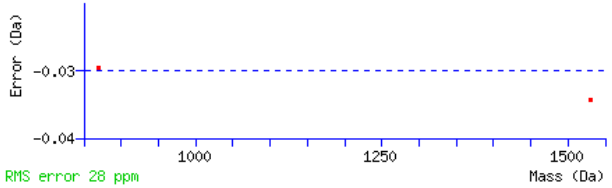


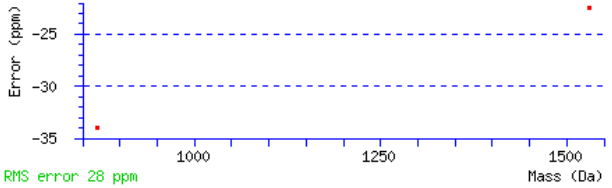

1042 ↑ PrP$^{+STS}$ Peroxiredoxin-4

```
  1 MEAPPPPPPL PATTLAPGRS RKLLLLPLLL FLLRAEAVRG FEAEERPRTR
 51 EEECHFYAGG QVYPGEVSRV SVAEHSLHLS KAKISKPAPY WEGTAVINGE
101 FKELKLTDYR GKYLVFFFYP LDFTFVCPTE IIAFGDRIDE FRSINTEVVA
151 CSVDSQFTHL AWINTPRRQG GLGSINIPLL ADLNHQISKD YGVYLEDSGH
201 TLRGLFIIDD KGILRQITLN DLPVGRSVDE TLRLVQAFQY TDKHGEVCPA
251 GWKPGSETII PDPAGKLKYF DKLN
```

| Query | Start - End | Observed | Mr(expt) | Mr(calc) | Delta | M | Score | Expect | Rank | U | Peptide |
|---|---|---|---|---|---|---|---|---|---|---|---|
| 106 | 190 - 203 | 542.2284 | 1623.6634 | 1623.7529 | -0.0895 | 0 | 5 | 2.4e+02 | 2 | U | K.DYGVYLEDSGHTLR.G |
| 107 | 190 - 203 | 812.8629 | 1623.7112 | 1623.7529 | -0.0417 | 0 | 13 | 37 | 1 | U | K.DYGVYLEDSGHTLR.G |
| 61 | 216 - 226 | 613.3267 | 1224.6388 | 1224.6826 | -0.0438 | 0 | 53 | 0.0043 | 1 | U | R.QITLNDLPVGR.S |
| 57 | 234 - 243 | 606.7949 | 1211.5752 | 1211.6186 | -0.0434 | 0 | 4 | 3.1e+02 | 3 | U | R.LVQAFQYTDK.H |
| 58 | 234 - 243 | 606.7957 | 1211.5768 | 1211.6186 | -0.0418 | 0 | 63 | 0.00041 | 1 | U | R.LVQAFQYTDK.H |
| 59 | 234 - 243 | 606.7988 | 1211.5830 | 1211.6186 | -0.0356 | 0 | 2 | 5e+02 | 2 | U | R.LVQAFQYTDK.H |

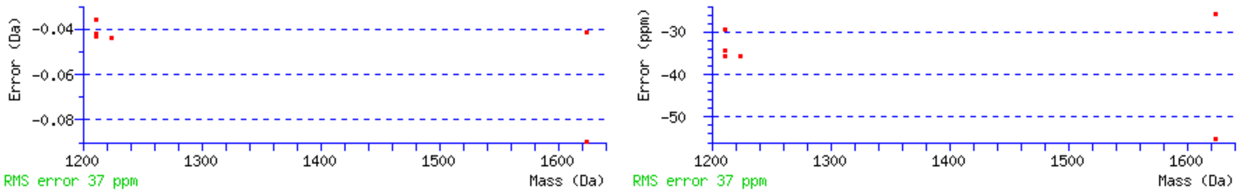


1016 ↑ PrP$^{+STS}$ Endoplasmic reticulum resident protein 29

```
  1 MAAAVPRAAF LSPLLPLLLG FLLLSAPHGG SGLHTKGALP LDTVTFYKVI
 51 PKSKFVLVKF DTQYPYGEKQ DEFKRLAENS ASSDDLLVAE VGISDYGDKL
101 NMELSEKYKL DKESYPVFYL FRDGDFENPV PYTGAVKVGA IQRWLKGQGV
151 YLGMPGCLPV YDALAGEFIR ASGVEARQAL LKQGQDNLSS VKETQKKWAE
201 QYLKIMGKIL DQGEDFPASE MTRIARLIEK NKMSDGKKEE LQKSLNILTA
251 FQKKGAEKEE L
```

| Query | Start – End | Observed | Mr(expt) | Mr(calc) | Delta | M | Score | Expect | Rank | U | Peptide |
|---|---|---|---|---|---|---|---|---|---|---|---|
| 114 | 37 – 48 | 662.8477 | 1323.6808 | 1323.7075 | -0.0266 | 0 | 73 | 4.6e-05 | 1 | U | K.GALPLDTVTFYK.V |
| 115 | 37 – 48 | 662.8478 | 1323.6810 | 1323.7075 | -0.0264 | 0 | 73 | 4.5e-05 | 1 | U | K.GALPLDTVTFYK.V |
| 116 | 37 – 48 | 662.8478 | 1323.6810 | 1323.7075 | -0.0264 | 0 | 72 | 4.9e-05 | 1 | U | K.GALPLDTVTFYK.V |
| 103 | 60 – 69 | 624.2736 | 1246.5326 | 1246.5506 | -0.0180 | 0 | 29 | 1.1 | 1 | U | K.FDTQYPYGEK.Q |
| 61 | 100 – 107 | 490.2320 | 978.4494 | 978.4692 | -0.0197 | 0 | 5 | 3.1e+02 | 2 | U | K.LNMELSEK.Y + Oxidation (M) |
| 140 | 123 – 137 | 804.8678 | 1607.7210 | 1607.7468 | -0.0257 | 0 | 30 | 0.8 | 1 | U | R.DGDFENPVPYTGAVK.V |
| 141 | 123 – 137 | 804.8709 | 1607.7272 | 1607.7468 | -0.0195 | 0 | 60 | 0.00074 | 1 | U | R.DGDFENPVPYTGAVK.V |
| 142 | 123 – 137 | 804.8713 | 1607.7280 | 1607.7468 | -0.0187 | 0 | 66 | 0.0002 | 1 | U | R.DGDFENPVPYTGAVK.V |
| 74 | 183 – 192 | 538.2585 | 1074.5024 | 1074.5305 | -0.0281 | 0 | 14 | 45 | 1 | U | K.QGQDNLSSVK.E |
| 148 | 209 – 223 | 862.8804 | 1723.7462 | 1723.7723 | -0.0261 | 0 | 81 | 4.8e-06 | 1 | U | K.ILDQGEDFPASEMTR.I + Oxidation (M) |
| 149 | 209 – 223 | 862.8806 | 1723.7466 | 1723.7723 | -0.0257 | 0 | 76 | 1.5e-05 | 1 | U | K.ILDQGEDFPASEMTR.I + Oxidation (M) |
| 150 | 209 – 223 | 862.8806 | 1723.7466 | 1723.7723 | -0.0257 | 0 | 79 | 8.8e-06 | 1 | U | K.ILDQGEDFPASEMTR.I + Oxidation (M) |
| 89 | 244 – 253 | 567.8157 | 1133.6168 | 1133.6444 | -0.0276 | 0 | 8 | 1.4e+02 | 1 | U | K.SLNILTAFQK.K |
| 90 | 244 – 253 | 567.8159 | 1133.6172 | 1133.6444 | -0.0272 | 0 | 8 | 1.5e+02 | 1 | U | K.SLNILTAFQK.K |

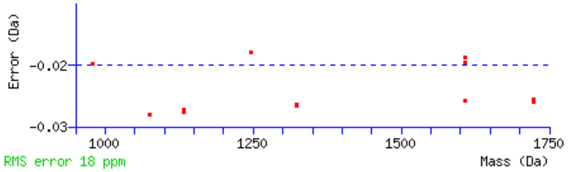


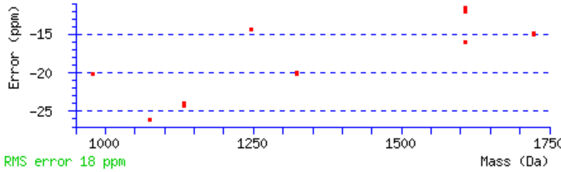


1105 ↓ PrP$^{+STS}$ Ubiquitin-conjugating enzyme E2 O

```
  1 MANIAVQRIK REFKEVLKSE ETSKNQIKVD LVDENFTELR GEIAGPPDTP
 51 YEGGRYQLEI KIPETYPFNP PKVRFITKIW HPNISSVTGA ICLDILKDQW
101 AAAMTLRTVL LSLQALLAAA EPDDPQDAVV ANQYKQNPEM FKQTARLWAH
151 VYAGAPVSSP EYTKKIENLC AMGFDRNAVI VALSSKSWDV ETATELLLSN
```

| Query | Start – End | Observed | Mr(expt) | Mr(calc) | Delta | M | Score | Expect | Rank | U | Peptide |
|---|---|---|---|---|---|---|---|---|---|---|---|
| 93 | 29 – 40 | 725.3582 | 1448.7018 | 1448.7147 | -0.0129 | 0 | 47 | 0.015 | 1 | U | K.VDLVDENFTELR.G |
| 106 | 62 – 74 | 519.9438 | 1556.8096 | 1556.8351 | -0.0255 | 1 | 0 | 7e+02 | 6 | U | K.IPETYPFNPPKVR.F |
| 89 | 166 – 176 | 671.2952 | 1340.5758 | 1340.5853 | -0.0094 | 0 | 19 | 9.7 | 1 | U | K.IENLCAMGFDR.N + Oxidation (M) |
| 64 | 177 – 186 | 501.2952 | 1000.5758 | 1000.5917 | -0.0158 | 0 | 1 | 9.2e+02 | 10 | U | R.NAVIVALSSK.S |
| 65 | 177 – 186 | 501.2959 | 1000.5772 | 1000.5917 | -0.0144 | 0 | 34 | 0.44 | 1 | U | R.NAVIVALSSK.S |

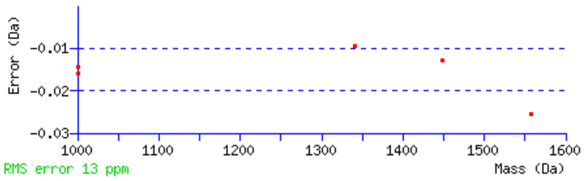


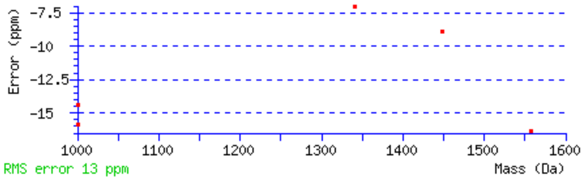

1161 ↓ $\text{PrP}^{+STS}$ Adenine phosphoribosyl transferase

```
  1 MADSELQLVE QRIRSFPDFP TPGVVFRDIS PVLKDPASFR AAIGLLARHL
 51 KATHGGRIDY IAGLDSRGFL FGPSLAQELG LGCVLIRKRG KLPGPTLWAS
101 YSLEYGKAEL EIQKDALEPG QRVVVVDDLL ATGGTMNAAC ELLGRLQAEV
151 LECVSLVELT SLKGREKLAP VPFFSLLQYE
```

| Query | Start – End | Observed | Mr(expt) | Mr(calc) | Delta | M | Score | Expect | Rank | U | Peptide |
|---|---|---|---|---|---|---|---|---|---|---|---|
| 100 | 15 – 27 | 733.3676 | 1464.7206 | 1464.7402 | -0.0195 | 0 | 51 | 0.0065 | 1 | U | R.SFPDFPTPGVVFR.D |
| 101 | 15 – 27 | 733.3709 | 1464.7272 | 1464.7402 | -0.0129 | 0 | 54 | 0.003 | 1 | U | R.SFPDFPTPGVVFR.D |
| 51 | 58 – 67 | 561.7784 | 1121.5422 | 1121.5717 | -0.0294 | 0 | 44 | 0.035 | 1 | U | R.IDYIAGLDSR.G |
| 52 | 58 – 67 | 561.7785 | 1121.5424 | 1121.5717 | -0.0292 | 0 | 37 | 0.21 | 1 | U | R.IDYIAGLDSR.G |
| 53 | 58 – 67 | 561.7856 | 1121.5566 | 1121.5717 | -0.0150 | 0 | 4 | 3.5e+02 | 2 | U | R.IDYIAGLDSR.G |
| 54 | 58 – 67 | 561.7857 | 1121.5568 | 1121.5717 | -0.0148 | 0 | 37 | 0.2 | 2 | U | R.IDYIAGLDSR.G |
| 30 | 115 – 122 | 443.2175 | 884.4204 | 884.4352 | -0.0147 | 0 | 6 | 2.6e+02 | 2 | U | K.DALEPGQR.V |
| 31 | 115 – 122 | 443.2177 | 884.4208 | 884.4352 | -0.0143 | 0 | 2 | 5.9e+02 | 3 | U | K.DALEPGQR.V |

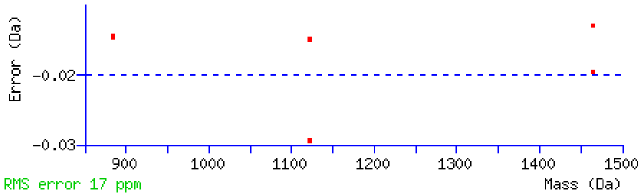


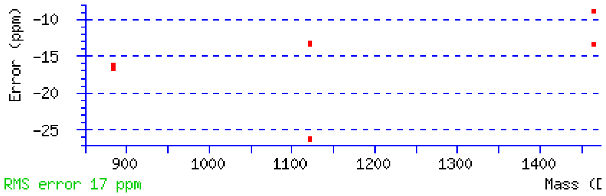


1157 ↑ $\text{PrP}^{+STS}$ Transgelin-2

```
  1 MANRGPAYGL SREVQQKIEK QYDADLEQIL IQWITTQCRK DVGRPQPGRE
 51 NFQNWLKDGT VLCELINGLY PEGQAPVKKI QASTMAFKQM EQISQFLQAA
101 ERYGINTTDI FQTVDLWEGK NMACVQRTLM NLGGLAVARD DGLFSGDPNW
151 FPKKSKENPR YFSDNQLQEG KNVIGLQMGT NRGASQAGMT GYGMPRQIL
```

| Query | Start – End | Observed | Mr (expt) | Mr (calc) | Delta | M | Score | Expect | Rank | U | Peptide |
|---|---|---|---|---|---|---|---|---|---|---|---|
| 13 | 5 – 12 | 410.6895 | 819.3644 | 819.4239 | -0.0594 | 0 | 34 | 0.44 | 1 | U | R.GPAYGLSR.E |
| 14 | 5 – 12 | 410.6898 | 819.3650 | 819.4239 | -0.0588 | 0 | 38 | 0.17 | 1 | U | R.GPAYGLSR.E |
| 31 | 41 – 49 | 491.2542 | 980.4938 | 980.5152 | -0.0213 | 0 | 11 | 67 | 1 | U | K.DVGRPQPGR.E |
| 32 | 41 – 49 | 491.2547 | 980.4948 | 980.5152 | -0.0203 | 0 | 17 | 16 | 1 | U | K.DVGRPQPGR.E |
| 39 | 50 – 57 | 539.7635 | 1077.5124 | 1077.5243 | -0.0119 | 0 | 29 | 1.3 | 1 | U | R.ENFQNWLK.D |
| 94 | 89 – 102 | 847.9014 | 1693.7882 | 1693.8093 | -0.0211 | 0 | 37 | 0.13 | 1 | U | K.QMEQISQFLQAAER.Y + Oxidation (M) |
| 95 | 89 – 102 | 847.9017 | 1693.7888 | 1693.8093 | -0.0205 | 0 | 28 | 1.1 | 1 | U | K.QMEQISQFLQAAER.Y + Oxidation (M) |
| 47 | 128 – 139 | 608.3370 | 1214.6594 | 1214.6805 | -0.0211 | 0 | 25 | 3.4 | 1 | U | R.TLMNLGGLAVAR.D |
| 48 | 128 – 139 | 608.3371 | 1214.6596 | 1214.6805 | -0.0209 | 0 | 9 | 1.2e+02 | 2 | U | R.TLMNLGGLAVAR.D |
| 52 | 128 – 139 | 616.3134 | 1230.6122 | 1230.6754 | -0.0632 | 0 | 92 | 5.3e-07 | 1 | U | R.TLMNLGGLAVAR.D + Oxidation (M) |
| 53 | 128 – 139 | 616.3135 | 1230.6124 | 1230.6754 | -0.0630 | 0 | 92 | 5.7e-07 | 1 | U | R.TLMNLGGLAVAR.D + Oxidation (M) |
| 54 | 128 – 139 | 616.3136 | 1230.6126 | 1230.6754 | -0.0628 | 0 | 92 | 5.7e-07 | 1 | U | R.TLMNLGGLAVAR.D + Oxidation (M) |
| 55 | 128 – 139 | 616.3300 | 1230.6454 | 1230.6754 | -0.0300 | 0 | 16 | 24 | 1 | U | R.TLMNLGGLAVAR.D + Oxidation (M) |
| 56 | 128 – 139 | 616.3303 | 1230.6460 | 1230.6754 | -0.0294 | 0 | 16 | 21 | 1 | U | R.TLMNLGGLAVAR.D + Oxidation (M) |
| 57 | 128 – 139 | 616.3345 | 1230.6544 | 1230.6754 | -0.0210 | 0 | 12 | 61 | 1 | U | R.TLMNLGGLAVAR.D + Oxidation (M) |
| 58 | 128 – 139 | 616.3349 | 1230.6552 | 1230.6754 | -0.0202 | 0 | 11 | 76 | 1 | U | R.TLMNLGGLAVAR.D + Oxidation (M) |
| 76 | 140 – 153 | 797.8456 | 1593.6766 | 1593.7100 | -0.0333 | 0 | 12 | 44 | 1 | U | R.DDGLFSGDPNWFPK.K |
| 77 | 140 – 153 | 797.8461 | 1593.6776 | 1593.7100 | -0.0323 | 0 | 31 | 0.55 | 1 | U | R.DDGLFSGDPNWFPK.K |
| 78 | 140 – 153 | 797.8494 | 1593.6842 | 1593.7100 | -0.0257 | 0 | 55 | 0.0022 | 1 | U | R.DDGLFSGDPNWFPK.K |
| 50 | 172 – 182 | 609.7947 | 1217.5748 | 1217.6187 | -0.0438 | 0 | 69 | 0.00013 | 1 | U | K.NVIGLQMGTNR.G + Oxidation (M) |
| 51 | 172 – 182 | 609.7950 | 1217.5754 | 1217.6187 | -0.0432 | 0 | 72 | 6.5e-05 | 1 | U | K.NVIGLQMGTNR.G + Oxidation (M) |

1293 ↓ $PrP^{+STS}$ 40S ribosomal protein S12

```
  1 MAEEGIAAGG VMDVNTALQE VLKTALIHDG LARGIREAAK ALDKRQAHLC
 51 VLASNCDEPM YVKLVEALCA EHQINLIKVD DNKKLGEWVG LCKIDREGKP
101 RKVVGCSCVV VKDYGKESQA KDVIEEYFKC KK
```

| Query | Start – End | Observed | Mr (expt) | Mr (calc) | Delta | M | Score | Expect | Rank | U | Peptide |
|---|---|---|---|---|---|---|---|---|---|---|---|
| 59 | 24 – 33 | 533.7844 | 1065.5542 | 1065.5931 | -0.0388 | 0 | 39 | 0.12 | 1 | U | K.TALIHDGLAR.G |
| 60 | 24 – 33 | 533.7858 | 1065.5570 | 1065.5931 | -0.0360 | 0 | 32 | 0.51 | 1 | U | K.TALIHDGLAR.G |
| 127 | 64 – 78 | 584.2962 | 1749.8668 | 1749.9447 | -0.0779 | 0 | 34 | 0.27 | 1 | U | K.LVEALCAEHQINLIK.V |
| 56 | 85 – 93 | 531.2506 | 1060.4866 | 1060.5376 | -0.0509 | 0 | 64 | 0.00047 | 1 | U | K.LGEWVGLCK.I |
| 57 | 85 – 93 | 531.2548 | 1060.4950 | 1060.5376 | -0.0425 | 0 | 69 | 0.00014 | 1 | U | K.LGEWVGLCK.I |
| 51 | 122 – 129 | 521.7330 | 1041.4514 | 1041.5019 | -0.0504 | 0 | 38 | 0.16 | 1 | U | K.DVIEEYFK.C |
| 52 | 122 – 129 | 521.7330 | 1041.4514 | 1041.5019 | -0.0504 | 0 | 38 | 0.17 | 1 | U | K.DVIEEYFK.C |

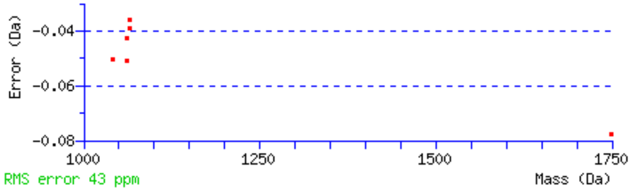


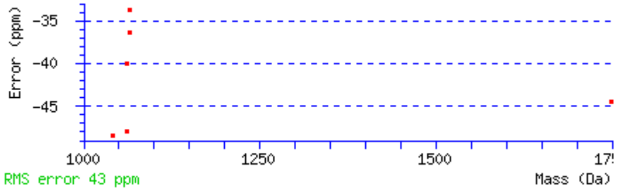

1294 ↓ PrP$^{+STS}$ Profilin-1

1 MAGWNAYIDN LMADGTCQDA AIVGYKDSPS VWAAVPGKTF VNITPAEVGV
51 LVGKDRSSFY VNGLTLGGQK CSVIRDSLLQ DGEFSMDLRT KSTGGAPTFN
101 VTVTKTDKTL VLLMGKEGVH GGLINKKCYE MASHLRRSQY

| Query | Start - End | Observed | Mr(expt) | Mr(calc) | Delta | M | Score | Expect | Rank | U | Peptide |
|---|---|---|---|---|---|---|---|---|---|---|---|
| 46 | 27 - 38 | 607.2922 | 1212.5698 | 1212.6139 | -0.0440 | 0 | 56 | 0.0022 | 1 | U | K.DSPSVWAAVPGK.T |
| 47 | 27 - 38 | 607.2926 | 1212.5706 | 1212.6139 | -0.0432 | 0 | 61 | 0.00071 | 1 | U | K.DSPSVWAAVPGK.T |
| 110 | 39 - 54 | 822.4415 | 1642.8684 | 1642.9294 | -0.0610 | 0 | 53 | 0.0039 | 1 | U | K.TFVNITPAEVGVLVGK.D |
| 111 | 39 - 54 | 822.4417 | 1642.8688 | 1642.9294 | -0.0606 | 0 | 57 | 0.0014 | 1 | U | K.TFVNITPAEVGVLVGK.D |
| 112 | 39 - 54 | 822.4454 | 1642.8762 | 1642.9294 | -0.0532 | 0 | 88 | 1.2e-06 | 1 | U | K.TFVNITPAEVGVLVGK.D |
| 114 | 39 - 56 | 639.0027 | 1913.9863 | 1914.0575 | -0.0712 | 1 | 63 | 0.00034 | 1 | U | K.TFVNITPAEVGVLVGKDR.S |
| 115 | 39 - 56 | 639.0027 | 1913.9863 | 1914.0575 | -0.0712 | 1 | 58 | 0.00095 | 1 | U | K.TFVNITPAEVGVLVGKDR.S |
| 103 | 76 - 89 | 813.3518 | 1624.6890 | 1624.7403 | -0.0512 | 0 | 8 | 1.3e+02 | 1 | U | R.DSLLQDGEFSMDLR.T |
| 104 | 76 - 89 | 813.3521 | 1624.6896 | 1624.7403 | -0.0506 | 0 | 12 | 49 | 1 | U | R.DSLLQDGEFSMDLR.T |
| 105 | 76 - 89 | 813.3521 | 1624.6896 | 1624.7403 | -0.0506 | 0 | 22 | 4.5 | 1 | U | R.DSLLQDGEFSMDLR.T |
| 106 | 76 - 89 | 813.3569 | 1624.6992 | 1624.7403 | -0.0410 | 0 | 27 | 1.4 | 1 | U | R.DSLLQDGEFSMDLR.T |
| 107 | 76 - 89 | 813.3569 | 1624.6992 | 1624.7403 | -0.0410 | 0 | 11 | 60 | 1 | U | R.DSLLQDGEFSMDLR.T |
| 108 | 76 - 89 | 821.3524 | 1640.6902 | 1640.7352 | -0.0450 | 0 | 24 | 3.2 | 1 | U | R.DSLLQDGEFSMDLR.T + Oxidation (M) |
| 109 | 76 - 89 | 821.3524 | 1640.6902 | 1640.7352 | -0.0450 | 0 | 1 | 5.9e+02 | 3 | U | R.DSLLQDGEFSMDLR.T + Oxidation (M) |
| 58 | 92 - 105 | 690.3169 | 1378.6192 | 1378.7093 | -0.0900 | 0 | 98 | 1.5e-07 | 1 | U | K.STGGAPTFNVTVTK.T |
| 59 | 92 - 105 | 690.3170 | 1378.6194 | 1378.7093 | -0.0898 | 0 | 78 | 1.4e-05 | 1 | U | K.STGGAPTFNVTVTK.T |
| 60 | 92 - 105 | 690.3171 | 1378.6196 | 1378.7093 | -0.0896 | 0 | 68 | 0.00014 | 1 | U | K.STGGAPTFNVTVTK.T |
| 48 | 106 - 116 | 609.8337 | 1217.6528 | 1217.7053 | -0.0525 | 1 | 77 | 2.1e-05 | 1 | U | K.TDKTLVLLMGK.E |
| 49 | 106 - 116 | 609.8341 | 1217.6536 | 1217.7053 | -0.0517 | 1 | 96 | 2.2e-07 | 1 | U | K.TDKTLVLLMGK.E |
| 50 | 106 - 116 | 617.8333 | 1233.6520 | 1233.7003 | -0.0482 | 1 | 1 | 6.9e+02 | 4 | U | K.TDKTLVLLMGK.E + Oxidation (M) |
| 14 | 109 - 116 | 437.7430 | 873.4714 | 873.5358 | -0.0643 | 0 | 29 | 1.5 | 1 | U | K.TLVLLMGK.E |
| 15 | 109 - 116 | 437.7430 | 873.4714 | 873.5358 | -0.0643 | 0 | 41 | 0.095 | 1 | U | K.TLVLLMGK.E |
| 16 | 109 - 116 | 437.7432 | 873.4718 | 873.5358 | -0.0639 | 0 | 29 | 1.5 | 1 | U | K.TLVLLMGK.E |
| 18 | 109 - 116 | 445.7539 | 889.4932 | 889.5307 | -0.0374 | 0 | 30 | 1.3 | 1 | U | K.TLVLLMGK.E + Oxidation (M) |
| 19 | 109 - 116 | 445.7540 | 889.4934 | 889.5307 | -0.0372 | 0 | 30 | 1.3 | 1 | U | K.TLVLLMGK.E + Oxidation (M) |
| 20 | 109 - 116 | 445.7540 | 889.4934 | 889.5307 | -0.0372 | 0 | 34 | 0.51 | 1 | U | K.TLVLLMGK.E + Oxidation (M) |
| 21 | 109 - 116 | 445.7547 | 889.4948 | 889.5307 | -0.0358 | 0 | 0 | 1.2e+03 | 2 | U | K.TLVLLMGK.E + Oxidation (M) |
| 38 | 117 - 127 | 576.3087 | 1150.6028 | 1150.6458 | -0.0430 | 1 | 30 | 0.99 | 1 | U | K.EGVHGGLINKK.C |
| 41 | 128 - 136 | 583.7365 | 1165.4584 | 1165.5008 | -0.0424 | 0 | 13 | 39 | 1 | U | K.CYEMASHLR.R |
| 42 | 128 - 136 | 583.7368 | 1165.4590 | 1165.5008 | -0.0418 | 0 | 3 | 4.5e+02 | 1 | U | K.CYEMASHLR.R |
| 43 | 128 - 136 | 583.7399 | 1165.4652 | 1165.5008 | -0.0356 | 0 | 17 | 18 | 1 | U | K.CYEMASHLR.R |
| 44 | 128 - 136 | 583.7401 | 1165.4656 | 1165.5008 | -0.0352 | 0 | 29 | 1.1 | 1 | U | K.CYEMASHLR.R |

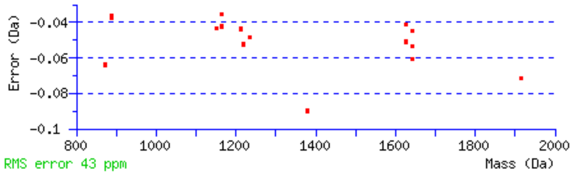


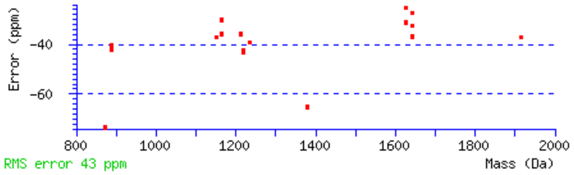

| | | | |
|---|---|---|---|
| PrP$^{+DMSO}$/ ctrl$^{+DMSO}$ | 1108 | ↑ PrP$^{+DMSO}$ | Ubiquitin-conjugating enzyme E2 K |

```
  1 MANIAVQRIK REFKEVLKSE ETSKNQIKVD LVDENFTELR GEIAGPPDTP
 51 YEGGRYQLEI KIPETYPFNP PKVRFITKIW HPNISSVTGA ICLDILKDQW
101 AAAMTLRTVL LSLQALLAAA EPDDPQDAVV ANQYKQNPEM FKQTARLWAH
151 VYAGAPVSSP EYTKKIENLC AMGFDRNAVI VALSSKSWDV ETATELLLSN
```

| Query | Start – End | Observed | Mr(expt) | Mr(calc) | Delta | M | Score | Expect | Rank | U | Peptide |
|---|---|---|---|---|---|---|---|---|---|---|---|
| 93 | 29 – 40 | 725.3582 | 1448.7018 | 1448.7147 | -0.0129 | 0 | 47 | 0.00086 | 1 | U | K.VDLVDENFTELR.G |
| 106 | 62 – 74 | 519.9438 | 1556.8096 | 1556.8351 | -0.0255 | 1 | 0 | 39 | 2 | U | K.IPETYPFNPPKVR.F |
| 89 | 166 – 176 | 671.2952 | 1340.5758 | 1340.5853 | -0.0094 | 0 | 19 | 0.55 | 1 | U | K.IENLCAMGFDR.N + Oxidation (M) |
| 64 | 177 – 186 | 501.2952 | 1000.5758 | 1000.5917 | -0.0158 | 0 | 1 | 50 | 2 | U | R.NAVIVALSSK.S |
| 65 | 177 – 186 | 501.2959 | 1000.5772 | 1000.5917 | -0.0144 | 0 | 34 | 0.024 | 1 | U | R.NAVIVALSSK.S |

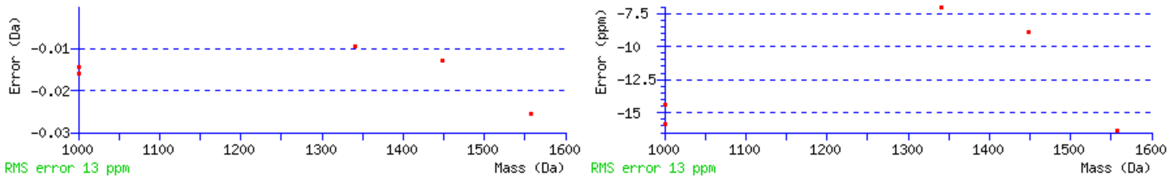


| | | | |
|---|---|---|---|
| | 1294 | ↑ PrP$^{+DMSO}$ | Profilin-1 |

```
  1 MAGWNAYIDN LMADGTCQDA AIVGYKDSPS VWAAVPGKTF VNITPAEVGV
 51 LVGKDRSSFY VNGLTLGGQK CSVIRDSLLQ DGEFSMDLRT KSTGGAPTFN
101 VTVTKTDKTL VLLMGKEGVH GGLINKKCYE MASHLRRSQY
```

| Query | Start – End | Observed | Mr(expt) | Mr(calc) | Delta | M | Score | Expect | Rank | U | Peptide |
|---|---|---|---|---|---|---|---|---|---|---|---|
| 46 | 27 – 38 | 607.2922 | 1212.5698 | 1212.6139 | -0.0440 | 0 | 56 | 0.0022 | 1 | U | K.DSPSVWAAVPGK.T |
| 47 | 27 – 38 | 607.2926 | 1212.5706 | 1212.6139 | -0.0432 | 0 | 61 | 0.00071 | 1 | U | K.DSPSVWAAVPGK.T |
| 110 | 39 – 54 | 822.4415 | 1642.8684 | 1642.9294 | -0.0610 | 0 | 53 | 0.0039 | 1 | U | K.TFVNITPAEVGVLVGK.D |
| 111 | 39 – 54 | 822.4417 | 1642.8688 | 1642.9294 | -0.0606 | 0 | 57 | 0.0014 | 1 | U | K.TFVNITPAEVGVLVGK.D |
| 112 | 39 – 54 | 822.4454 | 1642.8762 | 1642.9294 | -0.0532 | 0 | 88 | 1.2e-06 | 1 | U | K.TFVNITPAEVGVLVGK.D |
| 114 | 39 – 56 | 639.0027 | 1913.9863 | 1914.0575 | -0.0712 | 1 | 63 | 0.00034 | 1 | U | K.TFVNITPAEVGVLVGKDR.S |
| 115 | 39 – 56 | 639.0027 | 1913.9863 | 1914.0575 | -0.0712 | 1 | 58 | 0.00095 | 1 | U | K.TFVNITPAEVGVLVGKDR.S |
| 103 | 76 – 89 | 813.3518 | 1624.6890 | 1624.7403 | -0.0512 | 0 | 8 | 1.3e+02 | 1 | U | R.DSLLQDGEFSMDLR.T |
| 104 | 76 – 89 | 813.3521 | 1624.6896 | 1624.7403 | -0.0506 | 0 | 12 | 49 | 1 | U | R.DSLLQDGEFSMDLR.T |
| 105 | 76 – 89 | 813.3521 | 1624.6896 | 1624.7403 | -0.0506 | 0 | 22 | 4.5 | 1 | U | R.DSLLQDGEFSMDLR.T |
| 106 | 76 – 89 | 813.3569 | 1624.6992 | 1624.7403 | -0.0410 | 0 | 27 | 1.4 | 1 | U | R.DSLLQDGEFSMDLR.T |
| 107 | 76 – 89 | 813.3569 | 1624.6992 | 1624.7403 | -0.0410 | 0 | 11 | 60 | 1 | U | R.DSLLQDGEFSMDLR.T |
| 108 | 76 – 89 | 821.3524 | 1640.6902 | 1640.7352 | -0.0450 | 0 | 24 | 3.2 | 1 | U | R.DSLLQDGEFSMDLR.T + Oxidation (M) |
| 109 | 76 – 89 | 821.3524 | 1640.6902 | 1640.7352 | -0.0450 | 0 | 1 | 5.9e+02 | 3 | U | R.DSLLQDGEFSMDLR.T + Oxidation (M) |
| 58 | 92 – 105 | 690.3169 | 1378.6192 | 1378.7093 | -0.0900 | 0 | 98 | 1.5e-07 | 1 | U | K.STGGAPTFNVTVTK.T |
| 59 | 92 – 105 | 690.3170 | 1378.6194 | 1378.7093 | -0.0898 | 0 | 78 | 1.4e-05 | 1 | U | K.STGGAPTFNVTVTK.T |
| 60 | 92 – 105 | 690.3171 | 1378.6196 | 1378.7093 | -0.0896 | 0 | 68 | 0.00014 | 1 | U | K.STGGAPTFNVTVTK.T |
| 48 | 106 – 116 | 609.8337 | 1217.6528 | 1217.7053 | -0.0525 | 1 | 77 | 2.1e-05 | 1 | U | K.TDKTLVLLMGK.E |
| 49 | 106 – 116 | 609.8341 | 1217.6536 | 1217.7053 | -0.0517 | 1 | 96 | 2.2e-07 | 1 | U | K.TDKTLVLLMGK.E |
| 50 | 106 – 116 | 617.8333 | 1233.6520 | 1233.7003 | -0.0482 | 1 | 1 | 6.9e+02 | 4 | U | K.TDKTLVLLMGK.E + Oxidation (M) |
| 14 | 109 – 116 | 437.7430 | 873.4714 | 873.5358 | -0.0643 | 0 | 29 | 1.5 | 1 | U | K.TLVLLMGK.E |
| 15 | 109 – 116 | 437.7430 | 873.4714 | 873.5358 | -0.0643 | 0 | 41 | 0.095 | 1 | U | K.TLVLLMGK.E |
| 16 | 109 – 116 | 437.7432 | 873.4718 | 873.5358 | -0.0639 | 0 | 29 | 1.5 | 1 | U | K.TLVLLMGK.E |
| 18 | 109 – 116 | 445.7539 | 889.4932 | 889.5307 | -0.0374 | 0 | 30 | 1.3 | 1 | U | K.TLVLLMGK.E + Oxidation (M) |
| 19 | 109 – 116 | 445.7540 | 889.4934 | 889.5307 | -0.0372 | 0 | 30 | 1.3 | 1 | U | K.TLVLLMGK.E + Oxidation (M) |
| 20 | 109 – 116 | 445.7540 | 889.4934 | 889.5307 | -0.0372 | 0 | 34 | 0.51 | 1 | U | K.TLVLLMGK.E + Oxidation (M) |
| 21 | 109 – 116 | 445.7547 | 889.4948 | 889.5307 | -0.0358 | 0 | 0 | 1.2e+03 | 2 | U | K.TLVLLMGK.E + Oxidation (M) |
| 38 | 117 – 127 | 576.3087 | 1150.6028 | 1150.6458 | -0.0430 | 1 | 30 | 0.99 | 1 | U | K.EGVHGGLINKK.C |
| 41 | 128 – 136 | 583.7365 | 1165.4584 | 1165.5008 | -0.0424 | 0 | 13 | 39 | 1 | U | K.CYEMASHLR.R |
| 42 | 128 – 136 | 583.7368 | 1165.4590 | 1165.5008 | -0.0418 | 0 | 3 | 4.5e+02 | 1 | U | K.CYEMASHLR.R |
| 43 | 128 – 136 | 583.7399 | 1165.4652 | 1165.5008 | -0.0356 | 0 | 17 | 18 | 1 | U | K.CYEMASHLR.R |
| 44 | 128 – 136 | 583.7401 | 1165.4656 | 1165.5008 | -0.0352 | 0 | 29 | 1.1 | 1 | U | K.CYEMASHLR.R |

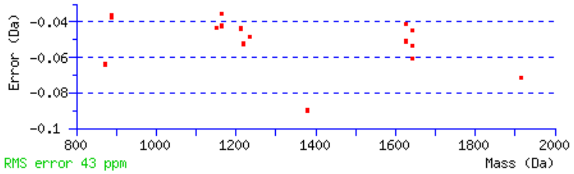


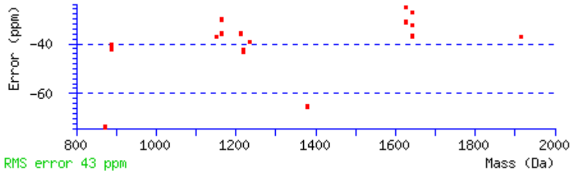


1161 ↓ PrP$^{+DMSO}$ Adenine phosphoribosyltransferase

```
  1 MADSELQLVE QRIRSFPDFP TPGVVFRDIS PVLKDPASFR AAIGLLARHL
 51 KATHGGRIDY IAGLDSRGFL FGPSLAQELG LGCVLIRKRG KLPGPTLWAS
101 YSLEYGKAEL EIQKDALEPG QRVVVVDDLL ATGGTMNAAC ELLGRLQAEV
151 LECVSLVELT SLKGREKLAP VPFFSLLQYE
```

| Query | Start – End | Observed | Mr(expt) | Mr(calc) | Delta | M | Score | Expect | Rank | U | Peptide |
|---|---|---|---|---|---|---|---|---|---|---|---|
| 100 | 15 – 27 | 733.3676 | 1464.7206 | 1464.7402 | -0.0195 | 0 | 51 | 0.0065 | 1 | U | R.SFPDFPTPGVVFR.D |
| 101 | 15 – 27 | 733.3709 | 1464.7272 | 1464.7402 | -0.0129 | 0 | 54 | 0.003 | 1 | U | R.SFPDFPTPGVVFR.D |
| 51 | 58 – 67 | 561.7784 | 1121.5422 | 1121.5717 | -0.0294 | 0 | 44 | 0.035 | 1 | U | R.IDYIAGLDSR.G |
| 52 | 58 – 67 | 561.7785 | 1121.5424 | 1121.5717 | -0.0292 | 0 | 37 | 0.21 | 1 | U | R.IDYIAGLDSR.G |
| 53 | 58 – 67 | 561.7856 | 1121.5566 | 1121.5717 | -0.0150 | 0 | 4 | 3.5e+02 | 2 | U | R.IDYIAGLDSR.G |
| 54 | 58 – 67 | 561.7857 | 1121.5568 | 1121.5717 | -0.0148 | 0 | 37 | 0.2 | 2 | U | R.IDYIAGLDSR.G |
| 30 | 115 – 122 | 443.2175 | 884.4204 | 884.4352 | -0.0147 | 0 | 6 | 2.6e+02 | 2 | U | K.DALEPGQR.V |
| 31 | 115 – 122 | 443.2177 | 884.4208 | 884.4352 | -0.0143 | 0 | 2 | 5.9e+02 | 3 | U | K.DALEPGQR.V |

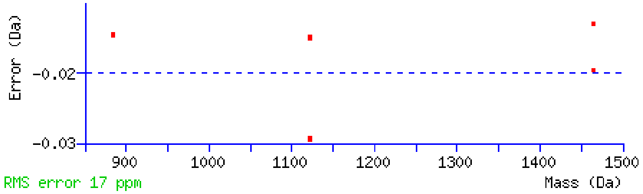


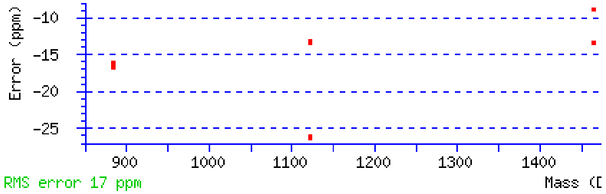


| | | | | |
|---|---|---|---|---|
| PrP$^{+DMSO}$/ ctrl$^{+STS}$ | - | - | - | - |
| ctrl$^{+STS}$/ ctrl$^{+DMSO}$ | 375 | ↓ ctrl$^{+STS}$ | Succinate dehydro - genase (ubiquinone) flavoprotein subunit, mitochondrial | 1 MSGVRGLSRL LSARRLALAK AWPTVLQTGT RGFHFTVDGN KRASAKVSDS<br>51 ISAQYPVVDH EFDAVVVGAG GAGLRAAFGL SEAGFNTACV TKLFPTRSHT<br>101 VAAQGGINAA LGNMEEDNWR WHFYDTVKGS DWLGDQDAIH YMTEQAPAAV<br>151 VELENYGMPF SRTEDGKIYQ RAFGGQSLKF GKGGQAHRCC CVADRTGHSL<br>201 LHTLYGRSLR YDTSYFVEYF ALDLLMENGE CRGVIALCIE DGSIHRIRAK<br>251 NTVVATGGYG RTYFSCTSAH TSTGDGTAMI TRAGLPCQDL EFVQFHPTGI<br>301 YGAGCLITEG CRGEGGILIN SQGERFMERY APVAKDLASR DVVSRSMTLE<br>351 IREGRGCGPE KDHVYLQLHH LPPEQLATRL PGISETAMIF AGVDVTKEPI<br>401 PVLPTVHYNM GGIPTNYKGQ VLRHVNGQDQ IVPGLYACGE AACASVHGAN<br>451 RLGANSLLDL VVFGRACALS IEESCRPGDK VPPIKPNAGE ESVMNLDKLR<br>501 FADGSIRTSE LRLSMQKSMQ NHAAVFRVGS VLQEGCGKIS KLYGDLKHLK<br>551 TFDRGMVWNT DLVETLELQN LMLCALQTIY GAEARKESRG AHAREDYKVR<br>601 IDEYDYSKPI QGQQKKPFEE HWRKHTLSYV DVGTGKVTLE YRPVIDKTLN<br>651 EADCATVPPA IRSY |

| Query | Start – End | Observed | Mr(expt) | Mr(calc) | Delta | M | Score | Expect | Rank | U | Peptide |
|---|---|---|---|---|---|---|---|---|---|---|---|
| 147 | 76 – 92 | 872.4245 | 1742.8344 | 1742.8298 | 0.0047 | 0 | 29 | 0.89 | 1 | U | R.AAFGLSEAGFNTACVTK.L |
| 61 | 251 – 261 | 547.7744 | 1093.5342 | 1093.5516 | -0.0174 | 0 | 12 | 51 | 2 | U | K.NTVVATGGYGR.T |
| 111 | 313 – 325 | 665.3373 | 1328.6600 | 1328.6684 | -0.0084 | 0 | 38 | 0.14 | 1 | U | R.GEGGILINSQGER.F |
| 112 | 313 – 325 | 665.3375 | 1328.6604 | 1328.6684 | -0.0080 | 0 | 32 | 0.55 | 1 | U | R.GEGGILINSQGER.F |
| 129 | 452 – 465 | 737.4243 | 1472.8340 | 1472.8351 | -0.0011 | 0 | 112 | 5.6e-09 | 1 | U | R.LGANSLLDLVVFGR.A |
| 131 | 452 – 465 | 737.4254 | 1472.8362 | 1472.8351 | 0.0011 | 0 | 7 | 1.7e+02 | 1 | U | R.LGANSLLDLVVFGR.A |
| 95 | 518 – 527 | 588.7869 | 1175.5592 | 1175.5506 | 0.0087 | 0 | 4 | 3.9e+02 | 2 | U | K.SMQNHAAVFR.V + Oxidation (M) |
| 106 | 625 – 636 | 638.8265 | 1275.6384 | 1275.6459 | -0.0075 | 0 | 2 | 6e+02 | 1 | U | K.HTLSYVDVGTGK.V |
| 136 | 648 – 662 | 814.4075 | 1626.8004 | 1626.8035 | -0.0031 | 0 | 47 | 0.016 | 1 | U | K.TLNEADCATVPPAIR.S |
| 137 | 648 – 662 | 814.4078 | 1626.8010 | 1626.8035 | -0.0025 | 0 | 6 | 2e+02 | 1 | U | K.TLNEADCATVPPAIR.S |

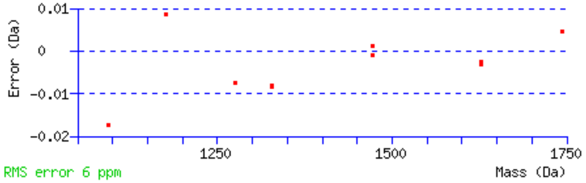


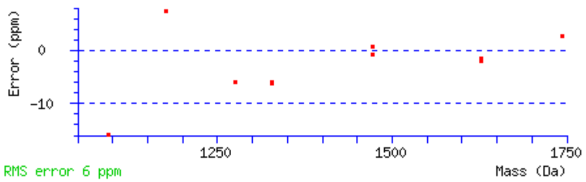


1108 ↑ ctrl$^{+STS}$ Ubiquitin-conjugating enzyme E2 K

```
  1 MANIAVQRIK REFKEVLKSE ETSKNQIKVD LVDENFTELR GEIAGPPDTP
 51 YEGGRYQLEI KIPETYPFNP PKVRFITKIW HPNISSVTGA ICLDILKDQW
101 AAAMTLRTVL LSLQALLAAA EPDDPQDAVV ANQYKQNPEM FKQTARLWAH
151 VYAGAPVSSP EYTKKIENLC AMGFDRNAVI VALSSKSWDV ETATELLLSN
```

| Query | Start – End | Observed | Mr(expt) | Mr(calc) | Delta | M | Score | Expect | Rank | U | Peptide |
|---|---|---|---|---|---|---|---|---|---|---|---|
| 93 | 29 – 40 | 725.3582 | 1448.7018 | 1448.7147 | -0.0129 | 0 | 47 | 0.00086 | 1 | U | K.VDLVDENFTELR.G |
| 106 | 62 – 74 | 519.9438 | 1556.8096 | 1556.8351 | -0.0255 | 1 | 0 | 39 | 2 | U | K.IPETYPFNPPKVR.F |
| 89 | 166 – 176 | 671.2952 | 1340.5758 | 1340.5853 | -0.0094 | 0 | 19 | 0.55 | 1 | U | K.IENLCAMGFDR.N + Oxidation (M) |
| 64 | 177 – 186 | 501.2952 | 1000.5758 | 1000.5917 | -0.0158 | 0 | 1 | 50 | 2 | U | R.NAVIVALSSK.S |
| 65 | 177 – 186 | 501.2959 | 1000.5772 | 1000.5917 | -0.0144 | 0 | 34 | 0.024 | 1 | U | R.NAVIVALSSK.S |

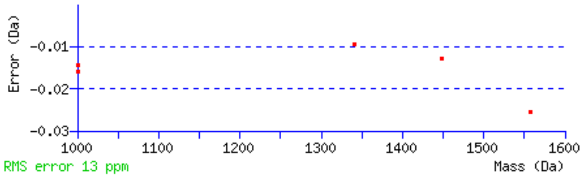


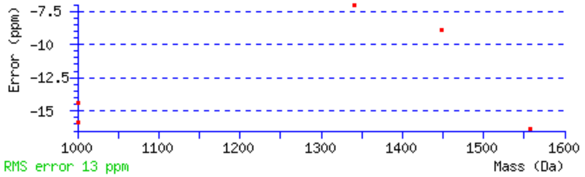

1161 ↓ ctrl$^{+STS}$ Adenine phosphoribosyltransferase

1 MADSELQLVE QRIRSFPDFP TPGVVFRDIS PVLKDPASFR AAIGLLARHL
51 KATHGGRIDY IAGLDSRGFL FGPSLAQELG LGCVLIRKRG KLPGPTLWAS
101 YSLEYGKAEL EIQKDALEPG QRVVVVDDLL ATGGTMNAAC ELLGRLQAEV
151 LECVSLVELT SLKGREKLAP VPFFSLLQYE

| Query | Start – End | Observed | Mr(expt) | Mr(calc) | Delta | M | Score | Expect | Rank | U | Peptide |
|---|---|---|---|---|---|---|---|---|---|---|---|
| 100 | 15 – 27 | 733.3676 | 1464.7206 | 1464.7402 | -0.0195 | 0 | 51 | 0.0065 | 1 | U | R.SFPDFPTPGVVFR.D |
| 101 | 15 – 27 | 733.3709 | 1464.7272 | 1464.7402 | -0.0129 | 0 | 54 | 0.003 | 1 | U | R.SFPDFPTPGVVFR.D |
| 51 | 58 – 67 | 561.7784 | 1121.5422 | 1121.5717 | -0.0294 | 0 | 44 | 0.035 | 1 | U | R.IDYIAGLDSR.G |
| 52 | 58 – 67 | 561.7785 | 1121.5424 | 1121.5717 | -0.0292 | 0 | 37 | 0.21 | 1 | U | R.IDYIAGLDSR.G |
| 53 | 58 – 67 | 561.7856 | 1121.5566 | 1121.5717 | -0.0150 | 0 | 4 | 3.5e+02 | 2 | U | R.IDYIAGLDSR.G |
| 54 | 58 – 67 | 561.7857 | 1121.5568 | 1121.5717 | -0.0148 | 0 | 37 | 0.2 | 2 | U | R.IDYIAGLDSR.G |
| 30 | 115 – 122 | 443.2175 | 884.4204 | 884.4352 | -0.0147 | 0 | 6 | 2.6e+02 | 2 | U | K.DALEPGQR.V |
| 31 | 115 – 122 | 443.2177 | 884.4208 | 884.4352 | -0.0143 | 0 | 2 | 5.9e+02 | 3 | U | K.DALEPGQR.V |

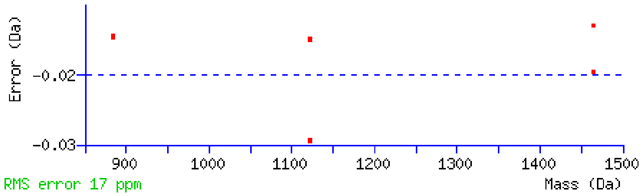


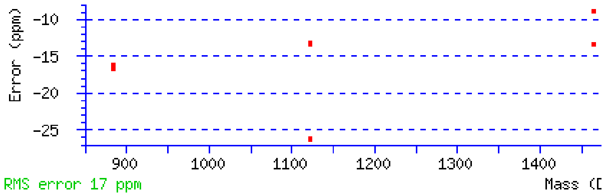


1294 ↑ ctrl$^{+STS}$ Profilin-1

1 MAGWNAYIDN LMADGTCQDA AIVGYKDSPS VWAAVPGKTF VNITPAEVGV
51 LVGKDRSSFY VNGLTLGGQK CSVIRDSLLQ DGEFSMDLRT KSTGGAPTFN
101 VTVTKTDKTL VLLMGKEGVH GGLINKKCYE MASHLRRSQY

| Query | Start - End | Observed | Mr(expt) | Mr(calc) | Delta | M | Score | Expect | Rank | U | Peptide |
|---|---|---|---|---|---|---|---|---|---|---|---|
| 46 | 27 - 38 | 607.2922 | 1212.5698 | 1212.6139 | -0.0440 | 0 | 56 | 0.0022 | 1 | U | K.DSPSVWAAVPGK.T |
| 47 | 27 - 38 | 607.2926 | 1212.5706 | 1212.6139 | -0.0432 | 0 | 61 | 0.00071 | 1 | U | K.DSPSVWAAVPGK.T |
| 110 | 39 - 54 | 822.4415 | 1642.8684 | 1642.9294 | -0.0610 | 0 | 53 | 0.0039 | 1 | U | K.TFVNITPAEVGVLVGK.D |
| 111 | 39 - 54 | 822.4417 | 1642.8688 | 1642.9294 | -0.0606 | 0 | 57 | 0.0014 | 1 | U | K.TFVNITPAEVGVLVGK.D |
| 112 | 39 - 54 | 822.4454 | 1642.8762 | 1642.9294 | -0.0532 | 0 | 88 | 1.2e-06 | 1 | U | K.TFVNITPAEVGVLVGK.D |
| 114 | 39 - 56 | 639.0027 | 1913.9863 | 1914.0575 | -0.0712 | 1 | 63 | 0.00034 | 1 | U | K.TFVNITPAEVGVLVGKDR.S |
| 115 | 39 - 56 | 639.0027 | 1913.9863 | 1914.0575 | -0.0712 | 1 | 58 | 0.00095 | 1 | U | K.TFVNITPAEVGVLVGKDR.S |
| 103 | 76 - 89 | 813.3518 | 1624.6890 | 1624.7403 | -0.0512 | 0 | 8 | 1.3e+02 | 1 | U | R.DSLLQDGEFSMDLR.T |
| 104 | 76 - 89 | 813.3521 | 1624.6896 | 1624.7403 | -0.0506 | 0 | 12 | 49 | 1 | U | R.DSLLQDGEFSMDLR.T |
| 105 | 76 - 89 | 813.3521 | 1624.6896 | 1624.7403 | -0.0506 | 0 | 22 | 4.5 | 1 | U | R.DSLLQDGEFSMDLR.T |
| 106 | 76 - 89 | 813.3569 | 1624.6992 | 1624.7403 | -0.0410 | 0 | 27 | 1.4 | 1 | U | R.DSLLQDGEFSMDLR.T |
| 107 | 76 - 89 | 813.3569 | 1624.6992 | 1624.7403 | -0.0410 | 0 | 11 | 60 | 1 | U | R.DSLLQDGEFSMDLR.T |
| 108 | 76 - 89 | 821.3524 | 1640.6902 | 1640.7352 | -0.0450 | 0 | 24 | 3.2 | 1 | U | R.DSLLQDGEFSMDLR.T + Oxidation (M) |
| 109 | 76 - 89 | 821.3524 | 1640.6902 | 1640.7352 | -0.0450 | 0 | 1 | 5.9e+02 | 3 | U | R.DSLLQDGEFSMDLR.T + Oxidation (M) |
| 58 | 92 - 105 | 690.3169 | 1378.6192 | 1378.7093 | -0.0900 | 0 | 98 | 1.5e-07 | 1 | U | K.STGGAPTFNVTVTK.T |
| 59 | 92 - 105 | 690.3170 | 1378.6194 | 1378.7093 | -0.0898 | 0 | 78 | 1.4e-05 | 1 | U | K.STGGAPTFNVTVTK.T |
| 60 | 92 - 105 | 690.3171 | 1378.6196 | 1378.7093 | -0.0896 | 0 | 68 | 0.00014 | 1 | U | K.STGGAPTFNVTVTK.T |
| 48 | 106 - 116 | 609.8337 | 1217.6528 | 1217.7053 | -0.0525 | 1 | 77 | 2.1e-05 | 1 | U | K.TDKTLVLLMGK.E |
| 49 | 106 - 116 | 609.8341 | 1217.6536 | 1217.7053 | -0.0517 | 1 | 96 | 2.2e-07 | 1 | U | K.TDKTLVLLMGK.E |
| 50 | 106 - 116 | 617.8333 | 1233.6520 | 1233.7003 | -0.0482 | 1 | 1 | 6.9e+02 | 4 | U | K.TDKTLVLLMGK.E + Oxidation (M) |
| 14 | 109 - 116 | 437.7430 | 873.4714 | 873.5358 | -0.0643 | 0 | 29 | 1.5 | 1 | U | K.TLVLLMGK.E |
| 15 | 109 - 116 | 437.7430 | 873.4714 | 873.5358 | -0.0643 | 0 | 41 | 0.095 | 1 | U | K.TLVLLMGK.E |
| 16 | 109 - 116 | 437.7432 | 873.4718 | 873.5358 | -0.0639 | 0 | 29 | 1.5 | 1 | U | K.TLVLLMGK.E |
| 18 | 109 - 116 | 445.7539 | 889.4932 | 889.5307 | -0.0374 | 0 | 30 | 1.3 | 1 | U | K.TLVLLMGK.E + Oxidation (M) |
| 19 | 109 - 116 | 445.7540 | 889.4934 | 889.5307 | -0.0372 | 0 | 30 | 1.3 | 1 | U | K.TLVLLMGK.E + Oxidation (M) |
| 20 | 109 - 116 | 445.7540 | 889.4934 | 889.5307 | -0.0372 | 0 | 34 | 0.51 | 1 | U | K.TLVLLMGK.E + Oxidation (M) |
| 21 | 109 - 116 | 445.7547 | 889.4948 | 889.5307 | -0.0358 | 0 | 0 | 1.2e+03 | 2 | U | K.TLVLLMGK.E + Oxidation (M) |
| 38 | 117 - 127 | 576.3087 | 1150.6028 | 1150.6458 | -0.0430 | 1 | 30 | 0.99 | 1 | U | K.EGVHGGLINKK.C |
| 41 | 128 - 136 | 583.7365 | 1165.4584 | 1165.5008 | -0.0424 | 0 | 13 | 39 | 1 | U | K.CYEMASHLR.R |
| 42 | 128 - 136 | 583.7368 | 1165.4590 | 1165.5008 | -0.0418 | 0 | 3 | 4.5e+02 | 1 | U | K.CYEMASHLR.R |
| 43 | 128 - 136 | 583.7399 | 1165.4652 | 1165.5008 | -0.0356 | 0 | 17 | 18 | 1 | U | K.CYEMASHLR.R |
| 44 | 128 - 136 | 583.7401 | 1165.4656 | 1165.5008 | -0.0352 | 0 | 29 | 1.1 | 1 | U | K.CYEMASHLR.R |

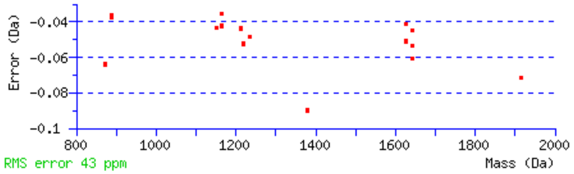


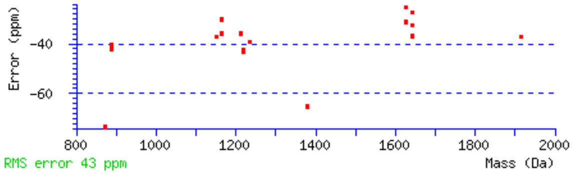

| | | | | |
|---|---|---|---|---|
| PrP$^{+STS}$/ PrP$^{+DMSO}$ | 384 | ↑ PrP$^{+STS}$ | Actin-interacting protein 1 | (see sequence and peptide matches below) |

```
  1 MPYEIKKVFA SLPQVERGVS KIVGGDPKGN SFLYTNGKCV ILRNIDNPAI
 51 ADIYTEHAHQ VVVAKYAPSG FYIASGDVSG KLRIWDTTQK EHLLKYEYQP
101 FAGKIKDIAW TEDSKRIAVV GEGREKFGAV FLWDSGSSVG EITGHNKVIN
151 SVDIKQSRPY RLVTGSDDNC AAFFEGPPFK FKFTISDHGR FVNCVRFSPD
201 GNRFATASAD GQIFIYDGKT GEKVCALGGS KAHDGGIYAI SWSPDSTHLL
251 SASGDKTSKI WDVNVNSVVN TFTMGSNVLD QQLGCLWQKD HLLSISLSGY
301 INYLDKNNPS KPLRVIKGHS KSIQCLTVHK NGGKSYIYSG SHDGHINYWD
351 SETGENDSFA GKGHTNQVSR MTVDEHGQLV SCSMDDTVRY TNLTLRDYSG
401 QGVVKLDVQP KCLAVGPGGY TVVVCIGQIV LLKDQRKCFS IDNPGYEPEV
451 VAVHPGGETV AVGGADGNVR LYSILGTTLK DEGKLLEAKG PVTDLAFSHD
501 GAFLAVCDAS KVVTVFSVAD GYSENNVFYG HHAKIVCLAW SPDNEHFASG
551 GMDMMVYVWT LSDPETRVKI QDAHRLHHVS SLAWLDEHTL VTTSHDASVK
601 EWTIAY
```

| Query | Start – End | Observed | Mr(expt) | Mr(calc) | Delta | M | Score | Expect | Rank | U | Peptide |
|---|---|---|---|---|---|---|---|---|---|---|---|
| 72 | 8 – 17 | 573.3154 | 1144.6162 | 1144.6241 | -0.0078 | 0 | 51 | 0.0014 | 1 | U | K.VFASLPQVER.G |
| 73 | 8 – 17 | 573.3155 | 1144.6164 | 1144.6241 | -0.0076 | 0 | 67 | 3.1e-05 | 1 | U | K.VFASLPQVER.G |
| 74 | 8 – 17 | 573.3158 | 1144.6170 | 1144.6241 | -0.0070 | 0 | 71 | 1.3e-05 | 1 | U | K.VFASLPQVER.G |
| 65 | 96 – 104 | 551.7612 | 1101.5078 | 1101.5131 | -0.0052 | 0 | 1 | 1.3e+02 | 1 | U | K.YEYQPFAGK.I |
| 55 | 397 – 405 | 476.7359 | 951.4572 | 951.4662 | -0.0089 | 0 | 4 | 62 | 1 | U | R.DYSGQGVVK.L |
| 66 | 471 – 480 | 554.8296 | 1107.6446 | 1107.6539 | -0.0093 | 0 | 2 | 99 | 1 | U | R.LYSILGTTLK.D |
| 67 | 471 – 480 | 554.8297 | 1107.6448 | 1107.6539 | -0.0091 | 0 | 3 | 68 | 1 | U | R.LYSILGTTLK.D |
| 94 | 471 – 484 | 513.2806 | 1536.8200 | 1536.8399 | -0.0199 | 1 | 8 | 20 | 1 | U | R.LYSILGTTLKDEGK.L |
| 95 | 471 – 484 | 513.2807 | 1536.8203 | 1536.8399 | -0.0196 | 1 | 5 | 36 | 1 | U | R.LYSILGTTLKDEGK.L |

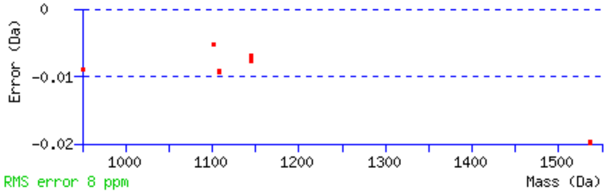


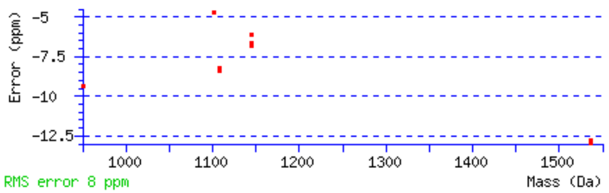


| | | | | |
|---|---|---|---|---|
| PrP$^{+STS}$/* ctrl$^{+DMSO}$ | - | - | - | |
