# Supplementary material for: Cellular prion protein mediates early apoptotic proteome alternation and phospho-modification in human neuroblastoma cells
Source: Cell Death Dis. 2017 Jan 19;8(1):e2557–. doi: 10.1038/cddis.2016.384 (PMC5386350; doi:10.1038/cddis.2016.384)

Groups compared	Spot no.	Protein ID	Accession no.
ctrl^{+STS}/ PrP^{+STS}	829	Inositol monophosphatase 1	

```
  1 MADPWQECMD YAVTLARQAG EVVCEAIKNE MNVMLKSSPV DLVTATDQKV
 51 EKMLISSIKE KYPSHSFIGE ESVAAGEKSI LTDNPTWIID PIDGTTNFVH
101 RFPFVAVSIG FAVNKKIEFG VVYSCVEGKM YTARKGKGAF CNGQKLQVSQ
151 QEDITKSLLV TELGSSRTPE TVRMVLSNME KLFCIPVHGI RSVGTAAVNM
201 CLVATGGADA YYEMGIHCWD VAGAGIIVTE AGGVLMDVTG GPFDLMSRRV
251 IAANNRILAE RIAKEIQVIP LQRDDED
```

Start - End	Observed	Mr(expt)	Mr(calc)	Delta	Miss	Sequence
157 - 167	581.3724	1160.7302	1160.6401	0.0902	0	K.SLLVTELGSSR.T
265 - 277	785.4480	1568.8814	1568.7682	0.1132	1	K.EIQVIPLQRDDED.-

MS/MS Fragmentation of **SLLVTELGSSR**

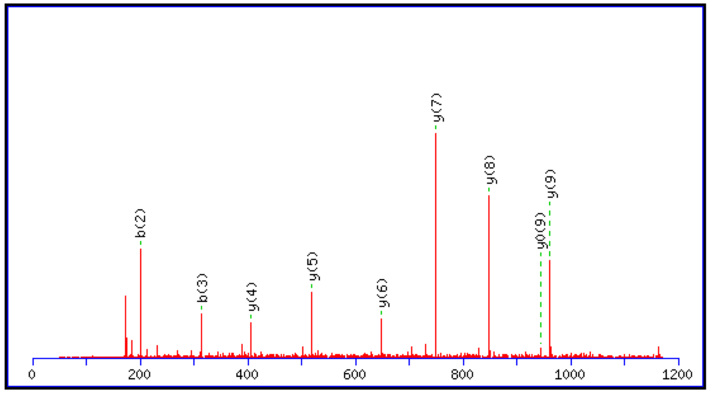

Monoisotopic mass of neutral peptide Mr(calc): 1160.6401
Ions Score: 50 **Expect:** 0.00069
Matches : 9/98 fragment ions using 12 most intense peaks (help)

Groups compared	Spot no.	Protein ID	Accession no.
	517	Calcium-binding mitochondrial carrier protein SCaMC-1	

```
  1 MAAGTLYTYP ENWRAFKALI AAQYSGAQVR VLSAPPHFHF GQTNRTPEFL
 51 RKFPAGKVPA FEGDDGFCVF ESNAIAYYVS NEELRGSTPE AAAQVVQWVS
101 FADSDIVPPA STWVFPTLGI MHHNKQATEN AKEEVRRILG LLDAYLKTRT
151 FLVGERVTLA DITVVCTLLW LYKQVLEPSF RQAFPNTNRW FLTCINQPQF
201 RAVLGEVKLC EKMAQFDAKK FAETQPKKDT PRKEKGSREE KQKPQAERKE
251 EKKAAAPAPE EEMDECEQAL AAEPKAKDPF AHLPKSTFVL DEFKRKYSNE
301 DTLSVALPYF WEHFDKDGWS LWYSEYRFPE ELTQTFMSCN LITGMFQRLD
351 KLRKNAFASV ILFGTNNSSS ISGVWVFRGQ ELAFPLSPDW QVDYESYTWR
401 KLDPGSEETQ TLVREYFSWE GAFQHVGKAF NQGKIFK
```

Start - End	Observed	Mr(expt)	Mr(calc)	Delta	Miss	Sequence
18 - 30	674.3998	1346.7850	1346.7306	0.0544	0	K.ALIAAQYSGAQVR.V
174 - 181	488.2858	974.5570	974.5185	0.0385	0	K.QVLEPSFR.Q
182 - 189	474.2512	946.4878	946.4621	0.0258	0	R.QAFPNTNR.W
286 - 295	621.3679	1240.7212	1240.6452	0.0761	1	K.STFVLDEFKR.K
402 - 414	722.8948	1443.7750	1443.7205	0.0545	0	K.LDPGSEETQTLVR.E

MS/MS Fragmentation of **STFVLDEFKR**

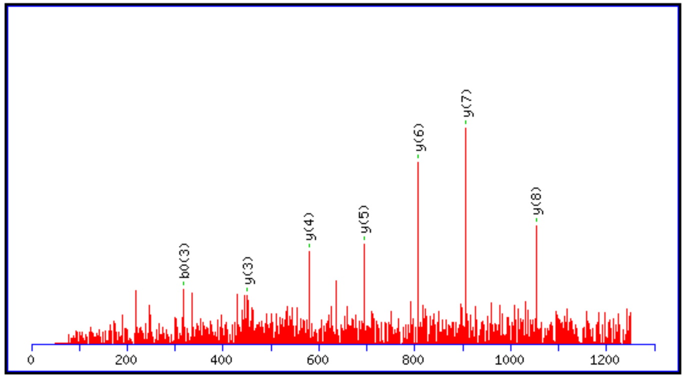

Monoisotopic mass of neutral peptide Mr(calc): 1240.6452
Ions Score: 34 **Expect:** 0.024
Matches : 7/86 fragment ions using 12 most intense peaks (help)

---

ctrl^{+DMSO}/ PrP^{+DMSO} | 646 | Stomatin-like protein 2

```
  1 MLARAARGTG ALLLRGSLLA SGRAPRRASS GLPRNTVVLF VPQQEAWVVE
 51 RMGRFHRILE PGLNILIPVL DRIRYVQSLK EIVINVPEQS AVTLDNVTLQ
101 IDGVLYLRIM DPYKASYGVE DPEYAVTQLA QTTMRSELGK LSLDKVFRER
151 ESLNASIVDA INQAADCWGI RCLRYEIKDI HVPPRVKESM QMQVEAERRK
201 RATVLESEGT RESAINVAEG KKQAQILASE AEKAEQINQA AGEASAVLAK
251 AKAKAEAIRI LAAALTQHNG DAAASLTVAE QYVSAFSKLA KDSNTILLPS
301 NPGDVTSMVA QAMGVYGALT KAPVPGTPDS LSSGSSRDVQ GTDASLDEEL
351 DRVKMS
```

Start - End	Observed	Mr(expt)	Mr(calc)	Delta	Miss	Sequence
212 - 221	509.2938	1016.5730	1016.5138	0.0592	0	R.ESAINVAEGK.K
212 - 221	509.2950	1016.5754	1016.5138	0.0616	0	R.ESAINVAEGK.K
212 - 222	573.3529	1144.6912	1144.6088	0.0825	1	R.ESAINVAEGKK.Q
223 - 233	594.3518	1186.6890	1186.6193	0.0697	0	K.QAQILASEAEK.A
223 - 233	594.3568	1186.6990	1186.6193	0.0797	0	K.QAQILASEAEK.A
234 - 250	835.9943	1669.9740	1669.8635	0.1106	0	K.AEQINQAAGEASAVLAK.A
234 - 250	835.9945	1669.9744	1669.8635	0.1110	0	K.AEQINQAAGEASAVLAK.A
322 - 337	757.9201	1513.8256	1513.7373	0.0884	0	K.APVPGTPDSLSSGSSR.D
322 - 337	757.9253	1513.8360	1513.7373	0.0988	0	K.APVPGTPDSLSSGSSR.D
322 - 337	757.9269	1513.8392	1513.7373	0.1020	0	K.APVPGTPDSLSSGSSR.D
338 - 352	831.9303	1661.8460	1661.7380	0.1080	0	R.DVQGTDASLDEELDR.V
338 - 352	831.9313	1661.8480	1661.7380	0.1100	0	R.DVQGTDASLDEELDR.V
338 - 354	630.6763	1889.0071	1888.9014	0.1057	1	R.DVQGTDASLDEELDRVK.M
338 - 354	630.6771	1889.0095	1888.9014	0.1081	1	R.DVQGTDASLDEELDRVK.M
338 - 354	630.6793	1889.0161	1888.9014	0.1147	1	R.DVQGTDASLDEELDRVK.M

MS/MS Fragmentation of **APVPGTPDSLSSGSSR**

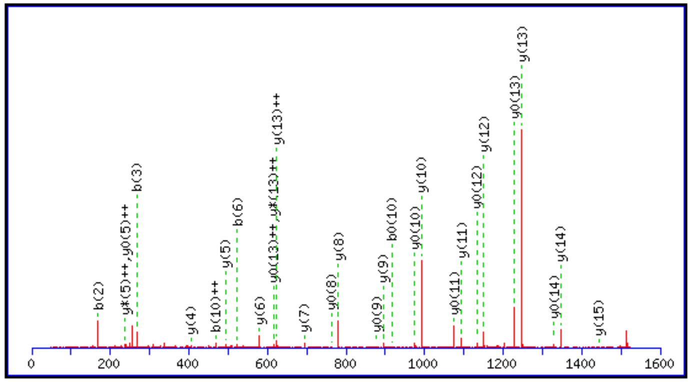

Monoisotopic mass of neutral peptide Mr(calc): 1513.7373
Ions Score: 88 **Expect:** 8.8e-08
Matches : 29/138 fragment ions using 45 most intense peaks (help)

390 Cystathionine beta-synthase

```
  1 MPSETPQAEV GPTGCPHRSG PHSAKGSLEK GSPEDKEAKE PLWIRPDAPS
 51 RCTWQLGRPA SESPHHHTAP AKSPKILPDI LKKIGDTPMV RINKIGKKFG
101 LKCELLAKCE FFNAGGSVKD RISLRMIEDA ERDGTLKPGD TIIEPTSGNT
151 GIGLALAAAV RGYRCIIVMP EKMSSEKVDV LRALGAEIVR TPTNARFDSP
201 ESHVGVAWRL KNEIPNSHIL DQYRNASNPL AHYDTTADEI LQQCDGKLDM
251 LVASVGTGGT ITGIARKLKE KCPGCRIIGV DPEGSILAEP EELNQTEQTT
301 YEVEGIGYDF IPTVLDRTVV DKWFKSNDEE AFTFARMLIA QEGLLCGGSA
351 GSTVAVAVKA AQELQEGQRC VVILPDSVRN YMTKFLSDRW MLQKGFLKEE
401 DLTEKKPWWW HLRVQELGLS APLTVLPTIT CGHTIEILRE KGFDQAPVVD
451 EAGVILGMVT LGNMLSSLLA GKVQPSDQVG KVIYKQFKQI RLTDTLGRLS
501 HILEMDHFAL VVHEQIQYHS TGKSSQRQMV FGVVTAIDLL NFVAAQERDQ
551 K
```

Start - End	Observed	Mr(expt)	Mr(calc)	Delta	Miss	Sequence	
84 - 91	452.7260	903.4374	903.4484	-0.0110	0	K.IGDTPMVR.I	Oxidation (M)
84 - 91	452.7267	903.4388	903.4484	-0.0096	0	K.IGDTPMVR.I	Oxidation (M)
84 - 91	452.7284	903.4422	903.4484	-0.0062	0	K.IGDTPMVR.I	Oxidation (M)
126 - 132	440.1901	878.3656	878.3804	-0.0147	0	R.MIEDAER.D	Oxidation (M)
197 - 209	496.2631	1485.7675	1485.7001	0.0674	0	R.FDSPESHVGVAWR.L	
360 - 369	565.2813	1128.5480	1128.5523	-0.0043	0	K.AAQELQEGQR.C	

MS/MS Fragmentation of **AAQELQEGQR**

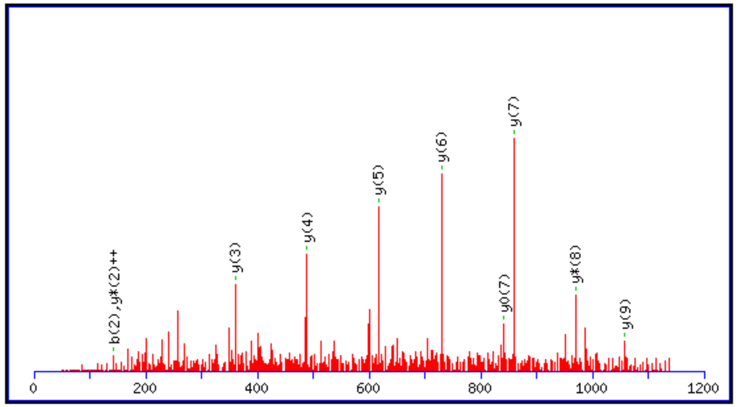

Monoisotopic mass of neutral peptide Mr(calc): 1128.5523
Ions Score: 50 **Expect:** 0.00061
Matches : 10/92 fragment ions using 11 most intense peaks (help)

355 T-complex protein 1

```
  1 MMGHRPVLVL SQNTKRESGR KVQSGNINAA KTIADIIRTC LGPKSMMKML
 51 LDPMGGIVMT NDGNAILREI QVQHPAAKSM IEISRTQDEE VGDGTTSVII
101 LAGEMLSVAE HFLEQQMHPT VVISAYRKAL DDMISTLKKI SIPVDISDSD
151 MMLNIINSSI TTKAISRWSS LACNIALDAV KMVQFEENGR KEIDIKKYAR
201 VEKIPGGIIE DSCVLRGVMI NKDVTHPRMR RYIKNPRIVL LDSSLEYKKG
251 ESQTDIEITR EEDFTRILQM EEEYIQQLCE DIIQLKPDVV ITEKGISDLA
301 QHYLMRANIT AIRRVRKTDN NRIARACGAR IVSRPEELRE DDVGTGAGLL
351 EIKKIGDEYF TFITDCKDPK ACTILLRGAS KEILSEVERN LQDAMQVCRN
401 VLLDPQLVPG GGASEMAVAH ALTEKSKAMT GVEQWPYRAV AQALEVIPRT
451 LIQNCGASTI RLLTSLRAKH TQENCETWGV NGETGTLVDM KELGIWEPLA
501 VKLQTYKTAV ETAVLLLRID DIVSGHKKKG DDQSRQGGAP DAGQE
```

Start - End	Observed	Mr(expt)	Mr(calc)	Delta	Miss	Sequence
79 - 85	426.2505	850.4864	850.4218	0.0646	0	K.SMIEISR.T Oxidation (M)
390 - 399	625.8340	1249.6534	1249.5543	0.0991	0	R.NLQDAMQVCR.N Carbamidomethyl (C); Oxidation (M)
428 - 438	677.3643	1352.7140	1352.6183	0.0957	0	K.AMTGVEQWPYR.A Oxidation (M)
450 - 461	667.4005	1332.7864	1332.6820	0.1045	0	R.TLIQNCGASTIR.L Carbamidomethyl (C)
450 - 461	667.4040	1332.7934	1332.6820	0.1115	0	R.TLIQNCGASTIR.L Carbamidomethyl (C)

MS/MS Fragmentation of TLIQNCGASTIR

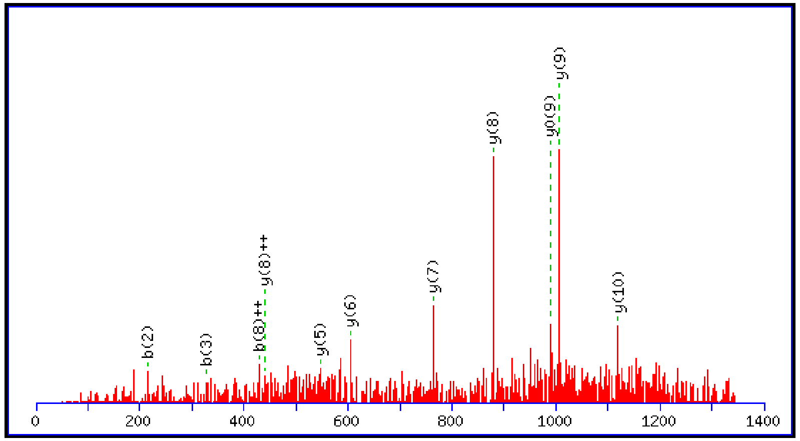

Monoisotopic mass of neutral peptide Mr(calc): 1332.6820
Variable modifications:
C6 : Carbamidomethyl (C)
Ions Score: 21 **Expect:** 0.51
Matches : 11/122 fragment ions using 26 most intense peaks (help)

ctrl^{+STS}/ PrP^{+DMSO}	1175	Rho GTPase-activating protein 1	MANHAPFETD ISTLTRFVME QGRKAQGTGE LTQLLNSLCT AIKAISSAVR<br>QAGIAQLYGI AGSTNVTGDQ VKKLDILSND LVINMLKSSY ATCVLVSEEN<br>TNAIIIEPEK RGKYVVCFDP LDGSSNIDCL VSIGTIFGIY RKKSTDEPSE<br>KDALQPGRDL VAAGYALYGS ATMLVLAMDC GVNCFMLDPS IGEFIMVDRD<br>VKMKKKGNIY SLNEGYAKDF DPAINEYLQR KKFPPDGSAP YGARYVGSMV<br>ADIHRTLVYG GIFLYPANKK SPSGKLRLLY ECNPIAYVME KAGGLATTGD<br>KDILDIVPTE IHQKAPVVMG SSEDVQEFLE IYRKHKAK

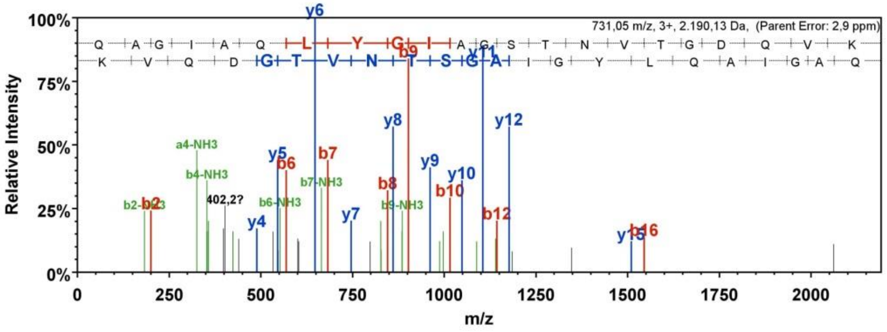

ctrl^{+DMSO}/ ctrl^{+STS}	651	26S protesome non-ATPase regulatory subunit 13	see sequence below

```
MPELAVQKVV VHPLVLLSVV DHFNRIGKVG NQKRVVGVLL GSWQKKVLDV
SNSFAVPFDE DDKDDSVWFL DHDYLENMYG MFKKVNARER IVGWYHTGPK
LHKNDIAINE LMKRYCPNSV LVIIDVKPKD LGLPTEAYIS VEEVHDDGTP
TSKTFEHVTS EIGAEEAEEV GVEHLLRDIK DTTVGTLSQR ITNQVHGLKG
LNSKLLDIRS YLEKVASGKL PINHQIIYQL QDVFNLLPDA SLQEFVKAFY
LKTNDQMVVV YLASLIRSVV ALHNLINNKI ANRDAEKKEG QEKEESKKER
KDDKEKEKSD AAKKEEKKEK K
```

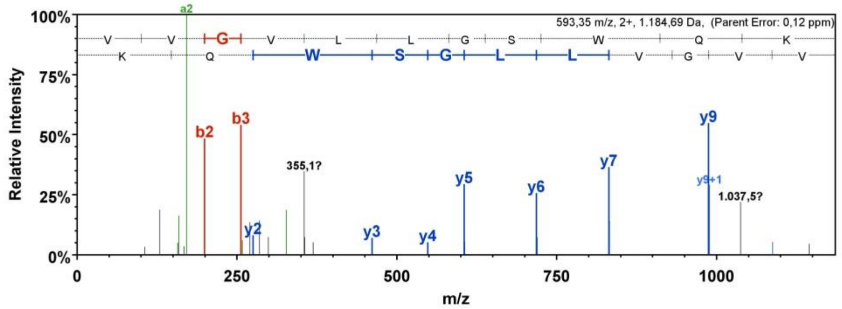

PrP^{+DMSO} / PrP^{+STS}	390	Cystathionine beta-synthase	see sequence below

```
  1 MPSETPQAEV GPTGCPHRSG PHSAKGSLEK GSPEDKEAKE PLWIRPDAPS
 51 RCTWQLGRPA SESPHHHTAP AKSPKILPDI LKKIGDTPMV RINKIGKKFG
101 LKCELLAKCE FFNAGGSVKD RISLRMIEDA ERDGTLKPGD TIIEPTSGNT
151 GIGLALAAAV RGYRCIIVMP EKMSSEKVDV LRALGAEIVR TPTNARFDSP
201 ESHVGVAWRL KNEIPNSHIL DQYRNASNPL AHYDTTADEI LQQCDGKLDM
251 LVASVGTGGT ITGIARKLKE KCPGCRIIGV DPEGSILAEP EELNQTEQTT
301 YEVEGIGYDF IPTVLDRTVV DKWFKSNDEE AFTFARMLIA QEGLLCGGSA
351 GSTVAVAVKA AQELQEGQRC VVILPDSVRN YMTKFLSDRW MLQKGFLKEE
401 DLTEKKPWWW HLRVQELGLS APLTVLPTIT CGHTIEILRE KGFDQAPVVD
451 EAGVILGMVT LGNMLSSLLA GKVQPSDQVG KVIYKQFKQI RLTDTLGRLS
501 HILEMDHFAL VVHEQIQYHS TGKSSQRQMV FGVVTAIDLL NFVAAQERDQ
551 K
```

Start - End	Observed	Mr(expt)	Mr(calc)	Delta	Miss	Sequence
84 - 91	452.7260	903.4374	903.4484	-0.0110	0	K.IGDTPMVR.I Oxidation (M)
84 - 91	452.7267	903.4388	903.4484	-0.0096	0	K.IGDTPMVR.I Oxidation (M)
84 - 91	452.7284	903.4422	903.4484	-0.0062	0	K.IGDTPMVR.I Oxidation (M)
126 - 132	440.1901	878.3656	878.3804	-0.0147	0	R.MIEDAER.D Oxidation (M)
197 - 209	496.2631	1485.7675	1485.7001	0.0674	0	R.FDSPESHVGVAWR.L
360 - 369	565.2813	1128.5480	1128.5523	-0.0043	0	K.AAQELQEGQR.C

MS/MS Fragmentation of **AAQELQEGQR**

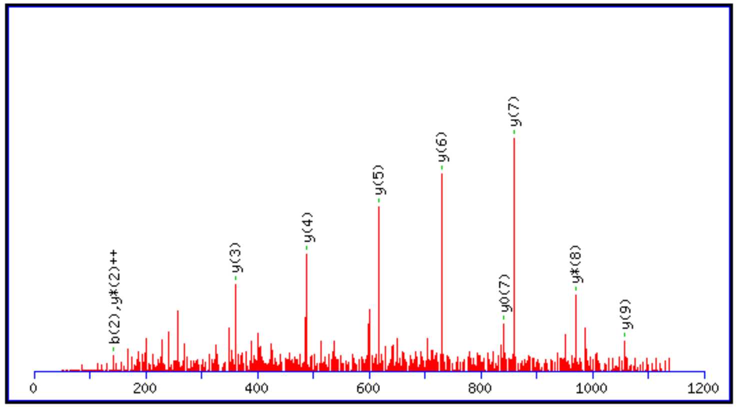

Monoisotopic mass of neutral peptide Mr(calc): 1128.5523
Ions Score: 50 **Expect:** 0.00061
Matches : 10/92 fragment ions using 11 most intense peaks (help)

ctrl+DMSO/ PrP+STS — 394 — unidentified — -

595 — Eukaryotic translation initiation factor 3 subunit I

1 MKPILLQGHE RSITQIKYNR EGDLLFTVAK DPIVNVWYSV NGERLGTYMG
51 HTGAVWCVDA DWDTKHVLTG SADNSCRLWD CETGKQLALL KTNSAVRTCG
101 FDFGGNIIMF STDKQMGYQC FVSFFDLR**DP SQIDNNEPYM K**IPCNDSKIT
151 SAVWGPLGEC IIAGHESGEL NQYSAK**SGEV LVNVKEHSRQ INDIQLSRDM**
201 **TMFVTASK**DN TAK**LFDSTTL EHQK**TFRTER PVNSAALSPN YDHVVLGGGQ
251 EAMDVTTTST RIGKFEARFF HLAFEEEFGR VK**GHFGPINS VAFHPDGKSY**
301 **SSGGEDGYVR** IHYFDPQYFE FEFEA

Start - End	Observed	Mr(expt)	Mr(calc)	Delta	Miss	Sequence
129 - 141	783.9001	1565.7856	1565.6668	0.1189	0	R.DPSQIDNNEPYMK.I Oxidation (M)
177 - 185	472.8097	943.6048	943.5338	0.0710	0	K.SGEVLVNVK.E
177 - 185	472.8104	943.6062	943.5338	0.0724	0	K.SGEVLVNVK.E
177 - 189	485.2970	1452.8692	1452.7685	0.1007	1	K.SGEVLVNVKEHSR.Q
177 - 189	485.2972	1452.8698	1452.7685	0.1013	1	K.SGEVLVNVKEHSR.Q
190 - 198	543.8392	1085.6638	1085.5829	0.0809	0	R.QINDIQLSR.D
190 - 198	543.8395	1085.6644	1085.5829	0.0815	0	R.QINDIQLSR.D
190 - 198	543.8403	1085.6660	1085.5829	0.0831	0	R.QINDIQLSR.D
190 - 198	543.8413	1085.6680	1085.5829	0.0851	0	R.QINDIQLSR.D
199 - 208	581.8041	1161.5936	1161.5046	0.0890	0	R.DMTMFVTASK.D 2 Oxidation (M)
199 - 208	581.8053	1161.5960	1161.5046	0.0914	0	R.DMTMFVTASK.D 2 Oxidation (M)
199 - 208	581.8060	1161.5974	1161.5046	0.0928	0	R.DMTMFVTASK.D 2 Oxidation (M)
214 - 224	440.2566	1317.7480	1317.6565	0.0915	0	K.LFDSTTLEHQK.T
214 - 224	440.2577	1317.7513	1317.6565	0.0948	0	K.LFDSTTLEHQK.T
283 - 298	560.6545	1678.9417	1678.8216	0.1201	0	K.GHFGPINSVAFHPDGK.S

299 - 310	638.8230	1275.6314	1275.5368	0.0947	0	K.SYSSGGEDGYVR.I
299 - 310	638.8237	1275.6328	1275.5368	0.0961	0	K.SYSSGGEDGYVR.I
299 - 310	638.8248	1275.6350	1275.5368	0.0983	0	K.SYSSGGEDGYVR.I
299 - 310	638.8253	1275.6360	1275.5368	0.0993	0	K.SYSSGGEDGYVR.I

MS/MS Fragmentation of **SGEVLVNVKEHSR**

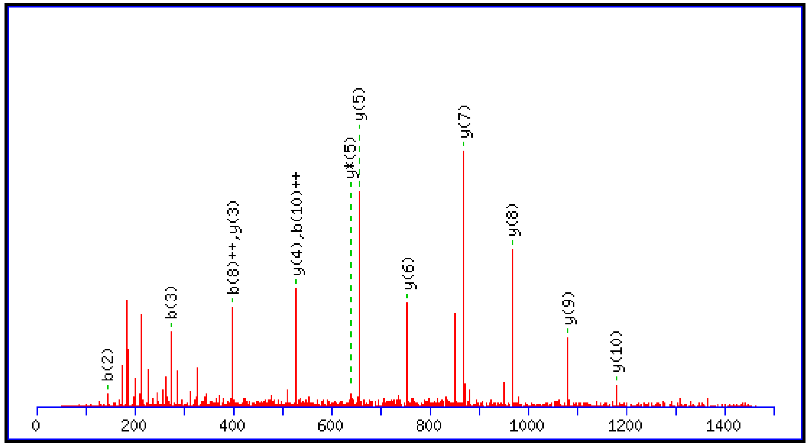

Monoisotopic mass of neutral peptide Mr(calc): 1452.7685
Ions Score: 65 **Expect:** 1.6e-05
Matches : 13/130 fragment ions using 15 most intense peaks (help)

474 40kDa peptidyl-prolyl-cis-trans isomerase

MAAAVPQRAW TVEQLRSEQL PKKDIIKFLQ DHGSDSFLAE HKLLGNIKNV
AKTANKDHLV NAYNHLFESK RFKGTETISK VSEQVKNVKL SDDKPKDSKS
EETLDEGPPK YTKSILKKGD KTNFPKKGDV VHCWYTGTLP DGTVFDTNIQ
TSSKKKKNAK PLSFKVGVGK VIRGWDEALL TMSKGEKARL EIEPEWAYGK
KGQPDAKIPP NTKLIFEVEL VDID

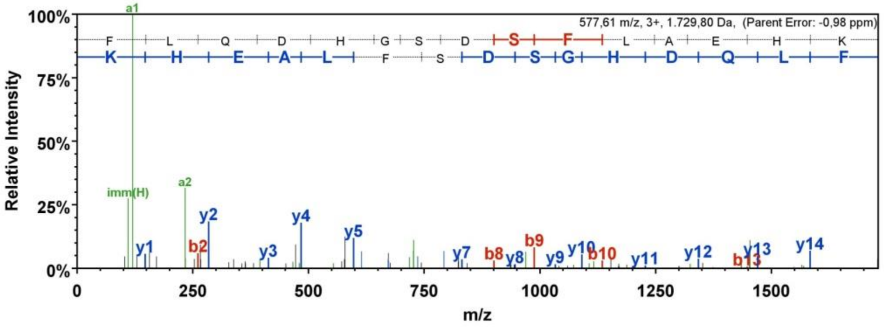

Supplement: Supplementary Figure 2 [file cddis2016384x2.pdf]
